# Validation of a Standard Luminescence Method for the Fast Determination of the Antimicrobial Activity of Nanoparticles in *Escherichia coli*

**DOI:** 10.3390/nano12132164

**Published:** 2022-06-23

**Authors:** Gonçalo A. Marcelo, Joana Galhano, Maria Paula Duarte, José Luis Capelo-Martínez, Carlos Lodeiro, Elisabete Oliveira

**Affiliations:** 1 BIOSCOPE Group, LAQV-REQUIMTE, Chemistry Department, NOVA School of Science and Technology, FCT NOVA, NOVA University Lisbon, 2829-516 Caparica, Portugal; goncalo.marcelo9@gmail.com (G.A.M.); j.galhano@campus.fct.unl.pt (J.G.); jlcm@fct.unl.pt (J.L.C.-M.); cle@fct.unl.pt (C.L.); 2 MEtRICs, NOVA School of Science and Technology, NOVA University Lisbon, 2829-516 Caparica, Portugal; 3 PROTEOMASS Scientific Society, Rua dos Inventores, Madam Parque, Caparica Campus, 2825-182 Caparica, Portugal

**Keywords:** nanoparticles, antimicrobial activity assessment, minimum inhibitory concentration, luminescence, *E. coli*

## Abstract

The use of nanoparticles in multiple industries has raised concerned voices about the assessment of their toxicity/antimicrobial activity and the development of standardized handling protocols. Issues emerge during the antimicrobial assaying of multiple cargo, colorimetric, colloidal nanoformulations, as standard protocols often rely on visual evaluations, or optical density (OD) measurements, leading to high variance inhibitory concentrations (MIC). Thus, a fast, luminescence-based assay for the effective assessment of the antimicrobial activity of nanoparticles is herein reported, using the bioluminescence of an in-house *E. coli* ATCC^®^ 8739^TM^ construct with the pMV306G13 + Lux plasmid (*E. coli* Lux). The new strain’s sensitivity to ofloxacin as a standard antibiotic was confirmed, and the methodology robustness verified against multiple nanoparticles and colorimetric drugs. The reduction of incubation from 24 to only 8 h, and the sole use of luminescence (LUX_490_) to accurately determine and distinguish MIC_50_ and MIC_90_, are two main advantages of the method. By discarding OD measurements, one can avoid turbidity and color interferences when calculating bacterial growth. This approach is an important tool that contributes to the standardization of methods, reducing samples’ background interference and focusing on luminescence as a direct probe for bacterial metabolic activity, growth and, most importantly, the correct assessment of nanomaterials’ antimicrobial activity.

## 1. Introduction

The growing use of nanomaterials and nanoparticles in multiple industries, from cosmetics to healthcare and environmental sectors, has given rise to concerned voices on the assessment of their toxicologic properties and the development of standardized and optimized experimental and clinical protocols when handling such materials [1,2,3]. One major player in the development of new nanoparticle-based applications is the pharmaceutical industry. This sector benefits from the former’s known high surface areas, carrier capacities, synergic properties, and antimicrobial activities to enhance the effectiveness of in-market drugs or develop completely new formulations capable of by-passing existing resistances, physical hindrances, and targeting specific targets in complex in vivo systems [4,5].

Notwithstanding, some issues emerge when nanometric colloidal formulations are used for such applications, specifically, during toxicological and antimicrobial assays. The standard protocols used to assess the toxicity and antimicrobial activity of a specific drug, or material, often rely on either the visual evaluation of the results, or on an optical density (OD)-dependent assessment of the samples [6], for the determination of the minimum inhibitory concentration (MIC), illustrating the concentration values at which ≥50% (MIC_50_) and ≥90% (MIC_90_) of the isolates in the test population are inhibited, respectively [7,8,9,10].

This is the case of the diffusion disk (agar dilution) technique or the Mueller–Hinton microdilution broth approach [6], commonly used in antimicrobial activity assays, which are prone to errors and high variability, especially when colloidal nanoparticles are involved. The fact that the first technique relies on the migration of target compounds from disks to the solid medium is itself a problem when working with nanoparticles, whose migration is often non-uniform, affinity- and size-dependent, as well as contact-dependent [11]. Moreover, the visual measurement of the resulting inhibition zones (halo diameters) is another factor accounting for the possible high variability of the results.

Regarding the second liquid-based approach, although solving all migration and material-bacteria contact problems, the fact that most protocols point to a visual determination of the MIC_50_ and the MIC_90_ encourages an erratic determination of both [12]. OD-mediated bacterial growth measurements (usually at 600 nm and 630 nm), although more sensible and in theory capable of more easily distinguishing between MIC_50_ and the MIC_90_ [11], are particularly hindered when nanoparticles are involved. The latter usually accounts for an increase in the turbidity of the evaluated medium, be it by their dispersion in it or for a time-promoted deposition at the bottom of the plate’s wells. This phenomenon results in erratic OD values with high standard deviations, decreasing the degree of certainty at which MIC_50_ and MIC_90_ are determined [13]. In summation, none of the above-mentioned methods is capable of providing direct information on bacteria metabolism and activity, with growth only being obtained by comparison with controls and further additional assays [14].

On the one hand, alternative colorimetric approaches have been developed to address these issues, as is the case of the well-established MTT/XTT reduction and Neutral Red uptake assays for cell toxicological/antimicrobial assays [15,16] or other reported bacterial staining-based assays [7,13,17]; on the other, their use with colorimetric nanoparticles (e.g., AuNPs, AgNPs, or iron oxide NPs) or cargo-filled porous nanoparticles (e.g., MCM-41 silica NPs) with colorimetric molecules/dyes, can still lead to baseline related aberrations and the wrong determination of both MIC_50_ and MIC_90_ [18]. In these cases, the colorimetric nanosystems can not only contribute to an increase in the baseline of the acquired absorbance spectra, but also interfere with the specific spectral maxima of each assay, thus producing inconsistent results.

This problem is especially aggravated when, for example, drug-delivery nanoparticles with multiple cargo, and multiple colorimetric profiles, are used, and the employment of a single assay is not sufficient. The development of multiple drug delivery nanoformulations has seen a consistent growth in the last decade, with particular interest in the production of multiple stimuli-responsive and multiple therapeutic materials for some of the most important health-related industries [19,20]. 

Bioluminescence is a widely known phenomenon that occurs in specific species and bacterial strains (e.g., *Mycobacterium marinum* and *Photorhabdus luminescens*). It results from the expression of luminescence responsible genes, without the need for external radiating or exciting sources [21]. The production of light by the action of luciferase enzymes on their cognate substrates can be directly correlated with the being’s metabolism, thus working as an immediate representative of its growth [22]. Bioluminescence-based assays to determine cell metabolic activity and/or growth have also been reported as highly sensitive, easy, and rapid methods [23,24,25,26]. The use of bioluminescence as a preferable tag in disease progression follow-up, as well as ecotoxicological, antimicrobial, and kinetic assays of molecular probes, has been reported in the literature to work as an excellent real-time probing mechanism with no disposition to interference [27,28,29,30,31,32]. Such an example is the use of luminescent *E. coli* for the detection of β-lactams [33] or as light-on/light-off sensors for bioavailable heavy metals, such as copper and mercury [34]. Interestingly, little effort has been made in the establishment of straightforward, standardized approaches fully devoted to the determination of the antimicrobial activity of nanoparticles and nanoformulations, where *in-lab* pre-modified strains or special genetic constructs are needed [35,36]. An example of such is the one-time reported use of luminescent *E. coli* strains on the investigation of copper nanoparticles’ possible antibacterial mechanisms [37].

Considering this, in this work we report a real-time, fast, luminescence-based antimicrobial assay for the effective assessment of the antimicrobial activity of multiple nanoformulations, and with no interference from the latter. The herein developed *state-of-the-art* approach uses the natural bioluminescence of an *in-house* constructed *Escherichia coli* strain (*E. coli* Lux), by insertion of the pMV306G13 + Lux plasmid in its parent commercial *Escherichia coli* ATCC^®^ 8739^TM^. This strain was tested for its sensitivity after the modification, and the methodology’s robustness was verified against multiple silica mesoporous nanoparticles, either bare or comprising a magnetic iron oxide core. A set of three loaded drugs was also used for its verification, applied in either single or dual formulations. Assay timings were also optimized by direct follow-up of the assayed bacteria. With this work, we seek to take a first step towards the establishment of a viable and standard solution for future clinical testing, and potential industrial applications of nanomaterials.

## 2. Materials and Methods

Iron(III) chloride hexahydrate (FeCl_3_·6H_2_O, 97.0–102.0%), iron(II) chloride hydrate (FeCl_2_·H_2_O, 99%), ammonium nitrate (NH_4_NO_3_, 99.999%), and tetraethyl orthosilicate (TEOS, ≥99.999% metals basis) were bought at AlfaAesar. *n*-Cetyltrimethylammonium bromide (CTAB), anhydrous N,N-dimethylformamide (DMF, 99.8%), anhydrous tetrahydrofuran (THF, 99.9%), acetone (≥99.5% GC), glucose monohydrate, and phosphate-buffered saline (PBS, tablets) were purchased from Sigma Aldrich. Ethanol absolute (EtOH, extra pure), dichloromethane (DCM, extra pure), and hydrochloric acid (HCl, 37%) were bought at Scharlab, SL. Dimethyl sulfoxide (DMSO) and methanol (MeOH) and sodium acetate anhydrous were acquired from CarloErba Reagents. Oleic acid (OA, 65.0–88.0%), ethanol 96%, sodium chloride (NaCl), and potassium chloride (KCl) were obtained from Honeywell Fluka. Sodium hydroxide (NaOH, tablets) was bought from Panreac AppliChem. Doxorubicin hydrochloride (DOX, >99%) and Epirubicin hydrochloride (EPI, >99%) were bought at LC Laboratories, while Ofloxacin (OFLO, ≥99%) and Kanamycin sulfate were purchased from Sigma-Aldrich, St. Louis, MO, USA. 

Bacto^TM^ Glycerol was bought from Becton Dickinson&Co. (Sparks, MD, USA), and magnesium chloride (MgCl_2_) was from LabChem Inc. Muller Hinton Agar (MHA), Muller Hinton Broth (MHB), Trypto Casein-Soy Agar (TSA) Trypto Casein-Soy Broth (TSB), tryptone, and yeast extract were all obtained from Biokar Diagnostics. All reagents were used as acquired, without any further purification, and all solutions, unless otherwise indicated, were prepared with deionized Millipore miliQ water.

Antibacterial assays were performed against Gram-negative *Escherichia coli* ATCC^®^ 8739^TM^ (*E. coli*). Transparent flat-bottom sterile 96-well plates were purchased from Greiner Bio-One and U-bottom opaque black 96-well plates from RatioLab (Dreieich-Buchschlag, Germany). pMV306G13 + Lux was a gift from Brian Robertson and Siouxsie Wiles (Addgene plasmid # 26160; http://n2t.net/addgene:26160 (accessed on 12 May 2022); RRID:Addgene_26160). GeneJET Plasmid Miniprep Kit #K0502 and all its respective solutions were used as bought from Thermo Scientific, Waltham, MA, USA.

DLS experiments were performed in a Malvern Nano ZS Zetasizer (633 nm laser diode), from the PROTEOMASS-BIOSCOPE Group LAQV-NOVAFCT. N_2_ adsorption-desorption isotherms were recorded in an ASAP 2010 automated analyzer (Micromeritics, Norcross, GA, USA), also from the Analysis Laboratory at LAQV-NOVAFCT. Specific surface areas were calculated from the adsorption data within the low-pressure range using the Brunauer–Emmett–Teller (BET) model. Pore size was determined following the Barrett–Joyner–Halenda (BJH) method. SEM images were obtained in a Quanta 650 FEG operating between 5–30 kV and 3.6 × 10^−4^ Pa of vacuum in the chamber. Transmission electron microscopy (TEM) images were obtained in a JEOL JEM-2100-HT operating at 200 kV, and TEM images were collected using a “OneView” 4k × 4k CCD camera, from INL-Braga.

UV–Vis absorption spectra were collected in a Jasco V-650 spectrophotometer and fluorescence spectroscopy measurements were taken in a HORIBA Scientific FLUOROMAX-4 spectrofluorometer, from the PROTEOMASS-BIOSCOPE Group LAQV-NOVAFCT. ATR-infrared spectra were collected in an Alpha II FT-IR spectrophotometer (Bruker, MA, USA), from the PROTEOMASS-BIOSCOPE Group LAQV-NOVAFCT. Magnetization studies were achieved in a Vibrating Sample Magnetometer (VSM) Lakeshore 7304 (Lake Shore Cryotronics, Westerville, OH, USA), with an applied magnetic field of 1.45 T, from the ISOM-Universidad Politécnica de Madrid.

Aseptic bacterial assays and modifications were handled in a Steril-VBH laminar flux chamber. Turbidity for each assayed bacterial suspension was adjusted in a DEN-1B McFarland Densiometer, from FCTNOVA (Grant-Bio). Incubations were conducted in a Mermmet Incubator B10, at 37 °C. Optical density (OD_600_, 600 nm) and luminescence (LUX_490_, 490 nm) measurements were conducted in a UV–Vis CLARIOstar^®^ Plus spectrophotometer, from the PROTEOMASS-BIOSCOPE Group LAQV-NOVAFCT (BMG Labtech, Ortenberg, Germany). Plasmid quantification was performed in a Nanodrop 1000 Spectrophotometer (Thermo Fisher). Electroporation protocols were performed in a Bio-Rad Gene Pulser^®^ from the Laboratory of Genetics of NOVA Medical School, Centro de Estudos de Doenças Crónicas (CEDOC).

### 2.1. Synthesis of Nanoparticles

#### 2.1.1. Mesoporous Silica Nanoparticles Synthesis

In a typical procedure, MCM-41 mesoporous silica nanoparticles (MNs) were synthesized via the Stöber method as reported in the literature [38,39]. Briefly, CTAB (180 mg) was dissolved in Milli-Q H_2_O (10 mL), at 50 °C. After 10 min, 20 mL of Milli-Q water (20 mL), ethylene glycol (10 mL), and NaOH 1M (700 μL) were sequentially added, and the mixture stirred for 30 min, at the same temperature. Afterwards, TEOS (750 μL) was added dropwise to the reactional mix and stirred for 2 h, at 70 °C. The material was collected by centrifugation and washed twice with MeOH, prior to dryness. 

#### 2.1.2. Oleic Acid Stabilized SPION Synthesis

Oleic acid stabilized super paramagnetic Fe_3_O_4_ magnetic nanoparticles (SPION@OA) were prepared by an adaptation of the co-precipitation method reported by Zhang et al. [40]. Briefly, FeCl_3_·6H_2_O (2.4 g) and FeCl_2_·4H_2_O (0.98 g) were vigorously dissolved in Milli-Q water (10 mL), under continuous Ar bubbling at 80 °C. After full dissolution, 5.0 mL of NH_4_OH (25 wt.%) was injected into the solution, which was stirred for 1 h at 80 °C, under Ar bubbling. Then, 425 μL of oleic acid (OA) was added and the reaction bubbled for an additional 5 min, before being sealed and stirred at 80 °C, for another 1.5 h. The resulting SPION-OA cores were magnetically decanted using a neodymium magnet and thoroughly washed with Milli-Q water until neutral. Excess OA was controlled by thin-layer chromatography. Later, SPION-OA were transferred in situ into chloroform (20 mL) to achieve a final particle concentration of 6.8 × 10^15^ particles/mL. The SPION concentration was calculated according to the total Fe content by ICP-AES and sizing by a TEM analysis.

#### 2.1.3. Single-Layer Mesoporous Silica Surface Modification

SPIONs’ surface modification with single-layer mesoporous silica (SPION@MNs) was achieved in an adept approach to that reported by Zhang et al. [40]. In brief, CTAB (150 mg) was dissolved in Milli-Q H_2_O (10 mL), mixed with SPION (0.74 mL), and sonicated for 1 h at 50 °C (35 kHz, degas mode). The resulting emulsion was then heated to 70 °C and stirred for 30 min to evaporate the residual chloroform. Then, Milli-Q H_2_O (30 mL), ethylene glycol (10 mL), and NH_4_OH (25 wt.%, 0.7 mL) were sequentially added to the mixture, which was stirred for another 30 min at 70 °C. After, TEOS (750 μL) was introduced dropwise to the reaction and stirred for 3 h, at 70 °C. The as-prepared sample was cooled to room temperature, recovered, and washed in MeOH 3 times and left to dry at 60 °C. SPION@MNs were structurally characterized by DLS, XRD, FTIR, N_2_ sorption, and SEM/TEM and magnetization properties confirmed by VSM.

#### 2.1.4. Template Removal 

The removal of the pores’ template, from both MNs and SPION@MNs, was achieved after resuspension in a 30 mg/mL NH_4_NO_3_ methanolic solution (50 mL) and stirring for 1 h at 60 °C. This process was repeated twice over, and the products obtained in the form of white and light brown magnetic powders, respectively, after washing in methanol and drainage at air [39].

#### 2.1.5. Drugs Loading and Release Assays

MNs and SPION@MNs materials were both single-loaded with OFLO, DOX, and EPI drugs, as well as with 1:1 dual combinatory cocktails of the same drugs (i.e., DOX + OFLO and EPI + OFLO). For single loadings, 5 mg of each material was resuspended in 2 mL of PBS buffer solution (pH 7.4, 0.01 M) with a drug concentration of 62.5 µg/mL. In total, 1:1 combinatory drug loadings were achieved by resuspending 5 mg of each material in 2 mL of each drug solution (62.5 µg/mL), in a PBS buffer, to a total volume of 4 mL. The resulting suspensions were stirred for 24 h at room temperature, in the dark. Each loaded material was recovered by centrifugation (10,000 rpm, 10 min) and washed 5 times with the PBS buffer solution. All supernatants were collected and quantified by UV–Vis spectroscopy, in triplicates, at 330 nm (OFLO, ε = 12,806 M^−1^ cm^−1^), 480 nm (EPI, ε = 12,958 M^−1^ cm^−1^), and 480 nm (DOX, ε = 10,096 M^−1^ cm^−1^). Loading efficiency (%E) and loading capacities (mg/g) were calculated by Equations (1) and (2).

(1) $$\%E=\frac{t_{drug}-f_{drug}}{t_{drug}}\times 100$$

(2) $$Loading\;capacity\;\left(\frac{\mathrm{mg}}{g}\right)=\frac{t_{drug\;}\left(\mathrm{mg}\right)-f_{drug\;}\left(\mathrm{mg}\right)}{m_{NP}\left(\mathrm{mg}\right)}$$

where *t_drug_* corresponds to the initial total mass of drug, *f_drug_* the final mass of drug in the supernatants, and *m_NP_* the mass of nanoparticles per loading batch of each material.

An in vitro drug release from loaded MNs and SPION@MNs was performed by suspending 0.5 mg of each material in 1.5 mL PBS pH 7.4 and PBS pH 4.0 solutions, accordingly. All suspensions were stirred at 37 °C, for 24 h, in the dark. After, the suspensions were centrifuged, and the supernatants were collected and quantified by the same approach.

### 2.2. Confirmation Assays

#### 2.2.1. CFU Counting

*E. coli* Lux was subcultured on TSA plates and incubated overnight at 37 °C. Isolated colonies were transferred to 0.85% NaCl, and the turbidity of the suspension was adjusted to 0.5 in the McFarland scale. From this suspension, serial decimal dilutions in 0.85% NaCl were performed and plated in TSA plates, that were incubated at 37 °C overnight. Finally, the number of visible colonies (CFU) present on the TSA plates was determined and multiplied by the dilution factor providing the CFU/mL of the initial suspension (Figure S2).

#### 2.2.2. Luminescence Assay

*E. coli* Lux was subcultured on TSA plates and incubated overnight at 37 °C. Isolated colonies were suspended in 100 µL 0.85% sterile NaCl. This suspension was transferred to a flat-bottom transparent 96-well plate, as well as to a U-bottom black opaque 96-well plate, for the acquisition of its OD_600_ intensity and LUX spectra, respectively. The LUX maximum intensity was determined for a 490 nm wavelength (LUX_490_).

#### 2.2.3. Optimization of Antimicrobial Assay Conditions with *E. coli* Lux

Bacterial suspensions of *E. coli* Lux and of the parental strain (*E. coli* ATCC^®^ 8739^TM^) were prepared as previously described (Section 2.2.2). Then, in sterile 96-well microplates, two-fold serial dilutions of ofloxacin (120 to 7.5 ng/mL) were prepared in sterile MHB to a final volume of 100 μL per well. Each well was inoculated with a 1:10 dilution of the previously prepared bacterial suspensions (to achieve a concentration of 10^6^ CFU/mL in each well). Negative controls (without ofloxacin) were also prepared. Plates were left to incubate at 37 °C, for appropriate timepoints, under both static and stirring (250 rpm) conditions. At 0, 1, 2, 3, 4, 6, 8, and 24 h, the OD_600_ was measured in a plate reader. The suspensions were then transferred to a black non-sterile 96-well plate and their LUX_490_ collected.

The susceptibility of *E. coli* Lux to OFLO was compared with that of its parental *E. coli* strain (Figure S3). An 8 h optimal assay time for the luminescent *E. coli* Lux transformant was determined by comparing OD_600_ and LUX_490_ vs. incubation time, with LUX_490_ being a direct measurement of metabolic activity and bacterial growth.

#### 2.2.4. Antimicrobial Activity Assessment

The antimicrobial activity of the different synthesized material was assessed against the parental *E. coli* strain and the new *E. coli* Lux strain.

In all assays, 2.5 mg/mL suspensions of all selected nanomaterials (MNS, SPION@MNs, MNs-drugs, and SPION@MNs-drugs) were prepared in H_2_O. Drug solutions containing the same concentration of the drugs loaded into each material were also prepared, in water. Bacterial suspensions (0.5 on the McFarland scale) were prepared as previously described in Section 2.2.2, and diluted with NaCl 0.85% to about 10^7^ CFU/mL. 

To evaluate and quantify the bacteriostatic effects of the tested nanocomposites, all samples were assayed by the broth microdilution method in a 96-well microplate. Briefly, two-fold serial dilutions of each sample, and controls, were prepared in sterile MHB to a final volume of 100 μL per well. Then, each well was inoculated with 10 μL of each previously prepared bacterial suspension (to achieve a concentration of 10^6^ CFU/mL in each well). Drug solutions containing the same concentration of the drugs loaded into each material were used as positive controls. Each material in the absence of bacteria and the bacteria without samples, were used as negative controls for the experiments. Plates regarding the *E. coli* Lux strain were left to incubate for 8 h, at 37 °C, whereas those of the parental *E. coli* were left to incubate for 24 h, at 37 °C.

OD_600_ and LUX_490_ measurements of the 96-well plates were conducted in a plate reader at 600 nm and 490 nm, respectively. Luminescence monitorization was achieved after transferring all suspensions into U-bottom opaque black 96-well plates. Bacterial growth and the MIC were calculated from luminescence data (Equation (3)), in the case of *E. coli* Lux, and from OD (Equation (4)), for the remaining bacteria.

(3) $$Bacterial\;Growth=\frac{Sample\;LUX_{490}}{Bacteria\;control\;LUX_{490}}$$

(4) $$Bacterial\;Growth=\frac{Sample\;OD_{600}-NP\;OD_{600}}{Bacteria\;control\;OD_{600}}$$

where *Sample OD*_600_ and *NP OD*_600_ stand for the incubated sample OD_600_ signal and the correspondent NP suspension OD_600_ control signal.

#### 2.2.5. Statistical Analysis

Data are presented as mean ± standard deviation, with *n* indicating the number of independent experiments. Student’s 2-tailed *t*-test was used to compare two groups with unequal variances, for α = 0.05. Statistical significance was shown as **** *p* < 0.0001, *** *p* < 0.001, ** *p* < 0.01, and * *p* < 0.05.

## 3. Results and Discussion

Bioluminescent bacteria transformants and an adapted Mueller–Hinton microdilution broth protocol were readily produced and validated, with reduced interference from the tested nanoformulations and increased sensitivity towards bacteria growth profiles, when compared to common protocols.

### 3.1. Bacterial Transformation and Validation

A parental commercially available *Escherichia coli* ATCC^®^ 8739^TM^, widely used to test the efficiency of antimicrobial agents, was selected as a *state-of-the-art* strain for bacterial transformation with the pMV306G13 + Lux plasmid (Figure S1) [28]. This plasmid, from *E. coli* DH5α, was selected as it contains the *Mycobacterium marinum* + *Photorhabdus luminescens* lux (luxABCDE) operon, capable of conferring constitutive bioluminescence to bacteria. The luxAB genes encode the production of the luciferase enzyme, while the luxCDE genes encode proteins that produce long-chain aldehydes, which are substrates for this bacterial luciferase [41]. Light production originates from a biochemical mechanism, catalyzed by the luciferase enzyme, in which FMNH_2_, the reduced form of the flavin mononucleotide, and an aliphatic aldehyde react in the presence of oxygen [42]. The transformation of the aldehyde into a long-chain fatty acid is accompanied by the production of flavin mononucleotide (FMN) and energy in the form of a photon of greenish-blue light, with an emission maximum at ca. 490 nm. The emission is readily detectable at naked-eye in a dark environment or by spectroscopic techniques [43]. The selected plasmid also contains a kanamycin resistance cassette that works as a selection marker for the new transformants of interest.

Plasmid purification, after extraction, is of extreme importance as extraction media’s high ionic strength can interfere with electroporation mechanisms [44]. The purification step yielded the plasmid at a concentration of 17.8 ng/mL and moderate purity. Purity was estimated according to the 260/280 nm ratio, with a ratio of ca. 1.8 being accepted as pure (obtained: 1.5) [45].

**Figure 1:**
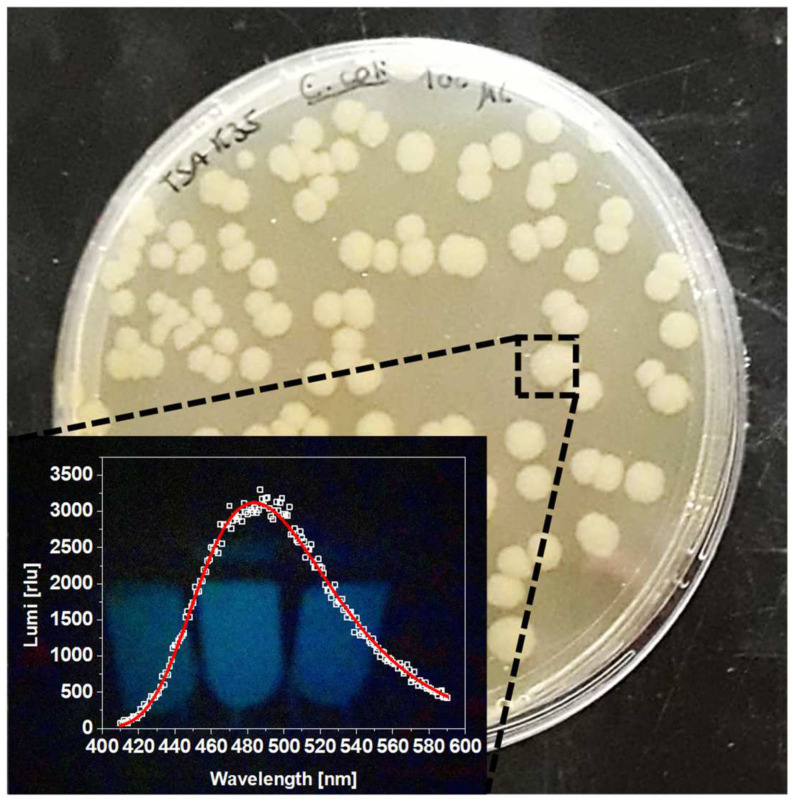
Bacterial transformants after electroporation; inset: *E. coli* LUX typic luminescence spectrum.

After competence induction and electroporation, the obtained transformants were left to recover and stabilize in a SOC medium and were then plated onto TSA-kanamycin plates. Kanamycin presence allowed for an appropriate selection of the transformants from the non-transformed kanamycin-susceptible parent *E. coli* strain. After 24 h of incubation, the plates presented several well-structured colonies, that when in the dark showed the expected intense greenish-blue luminescence, easily detected at naked-eye. Luminescence spectra of four random isolated colonies suspensions were spectroscopically scanned, with all showing a maximum of luminescence at 490 nm (LUX_490_) and high intensities of ca. 3000 relative luminescence units (rlu) (Figure 1). Thus, this confirmed a successful transformation and production of a luminescent *E. coli* Lux strain.

The relationship between the turbidity of the new *E. coli* Lux suspensions and cell concentration in CFU/mL was verified (Figure S2). This relationship is essential for antibacterial assays, since cell concentration is regulated by adjusting the turbidity of bacterial suspensions according to the McFarland scale, with a turbidity of 0.5 corresponding to a cell concentration of ca. 10^8^ CFU/mL [46,47]. As *E. coli* Lux transformants show intrinsic luminescence, it was necessary to verify if this phenomenon had no interference with the measurement of the turbidity of the sample, and subsequently with cell concentration. The preparation of successive decimal dilutions from an initial 0.5 McFarland suspension and further 24 h incubation, in TSA plates, confirmed the correct concentration of the initial suspension (ca. 10^8^ CFU/mL), proving that the bioluminescence of *E. coli* Lux has no interference in the turbidity of the suspensions and further adjustment of bacterial concentration.

### 3.2. E. coli Lux Susceptibility to Ofloxacin

Ofloxacin is a well-known second-generation fluoroquinolone with a wide antibiotic spectrum, used for the treatment of several types of infections in the respiratory tract, kidney, skin, soft tissue, and urinary tract [48]. This antibiotic is active against many Gram-positive bacteria and most Gram-negative bacteria, *E. coli* included, via its inhibitory action against DNA gyrases and type II bacterial topoisomerases [49].

**Figure 2:**
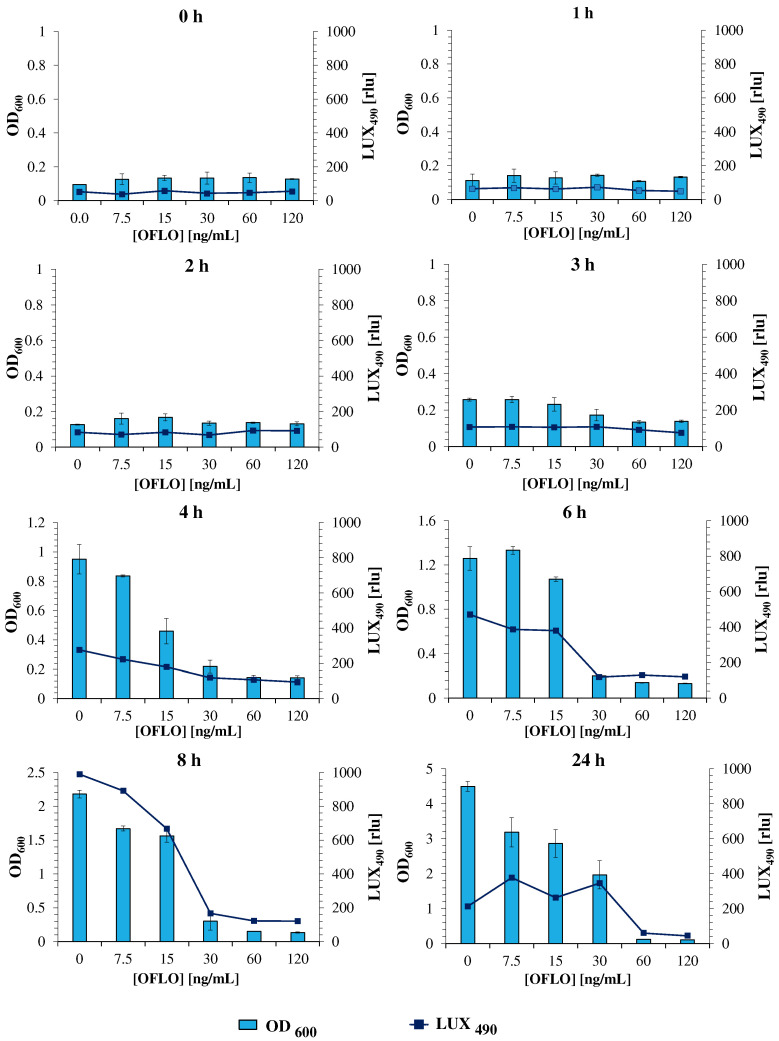
Temporal bacterial response of *E. coli* Lux to ofloxacin, under static conditions, for different concentrations. Responses were given in the form of LUX_490_ signal intensity and OD_600_.

The assessment of *E. coli* Lux susceptibility to ofloxacin, as a model antibiotic, is thus an essential hallmark to the validation of the new strain, as a similar response to that obtained for the parent *E. coli* is pursued. In this assay, *E. coli* Lux was incubated with a range of ofloxacin concentrations from 120 to 7.5 ng/mL, and its response (in OD_600_ and LUX_490_) was evaluated at different timepoints, under both static and orbital shaking incubation conditions. The assaying ofloxacin concentration range was set after an initial assay with the non-luminescent parent *E. coli* strain (OD_600_) to determine both the MIC_50_ and MIC_90_ of ofloxacin (Figure S3). From the initial static and shaking datasets, it was found that a more reliable positive correlation between OD_600_ and LUX_490_, and consequently a better estimation of bacterial metabolic activity and thus, growth, was obtained under static conditions (Figure S4). For this, static growth was deemed as the optimal mechanical condition for the method, and the data obtained under static growth were considered for further analysis (Figure 2). 

During the first 4 h, the lack of significative bioluminescence variations and its overall low intensity between different ofloxacin concentrations, following a parallel trend to those represented by OD_600_ measurements, confirmed that this period corresponded to the lag phase of bacterial growth, where no significant growth was evident, and no significant bacterial activity occurred [50]. However, after 6 h of incubation, and now in the exponential phase of bacterial growth, characterized by an exponential increase in bacterial growth and metabolic activity, an increase in the LUX_490_ of the bacteria exposed to 15 and 7.5 ng/mL ofloxacin pointed toward a less effective action of the drug against *E. coli* Lux in this range. This was further corroborated by the low LUX_490_ and OD_600_ measurements obtained for higher concentrations (120, 60, and 30 ng/mL), where ofloxacin action was evident and the intensity of bioluminescence and OD_600_ was kept minimal. The trend continued and was more evident after 8 h, where the LUX_490_ signal was maximum at an ofloxacin concentration of 7.5 ng/mL (ca. 890 rlu). At this point, the LUX_490_ signal tightly mimicked the behavior of OD_600_ measurements, allowing for the estimation of both inhibitory concentrations (MIC_50_ and MIC_90_) through a direct correlation between bacterial metabolic activity and LUX_490_ intensity and its fluctuations in response to several antibiotic concentrations. A direct look at the luminescence curve highlighted the disparity and abrupt decrease in the LUX_490_ signal between ofloxacin concentrations of 15 and 30 ng/mL, from 668 rlu to 170 rlu, representing a 75% reduction from one point to another. This reduction, supported by similar profiles obtained through OD_600_ measurements, allowed us to determine with certainty the presence of the MIC_50_ of ofloxacin for this bacterial strain.

Interestingly, the LUX_490_-determined MIC_50_ (between 15–30 ng/mL), at 8 h, was the same as that determined for the parent *E. coli* strain, via OD_600_ at 24 h (30 ng/mL of ofloxacin) (Figure S3) [11], verifying three major points. First, that the response of *E. coli* Lux to ofloxacin is similar to that of the wild-type parent *E. coli* strain; second, that it is possible to accurately determine inhibition concentrations, equivalent to those obtained by the standardized OD_600_ measurements after 18–24 h of incubation, through luminescence measurements after just 8 h of incubation; and lastly, that the results obtained for LUX_490_ are a reliable source for the estimation of bacterial metabolic activity and growth, in spite of OD_600_ measurements.

**Figure 3:**
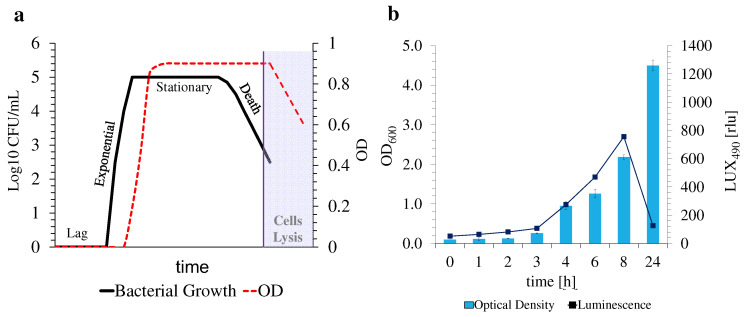
Typical bacterial growth curve and its correlation with OD measurements (**a**) adapted from [51]. Obtained bacterial growth curve and correlation with the recorded luminescence, emitted by luminescent *E. coli* Lux (**b**).

These last two points are of utter interest, as OD_600_ measurements solely rely on the turbidity of the samples, which is directly correlated to the quantity of cells in the suspension, regardless of their growth phase at the moment of the measurement. This means that dead bacterial cells continue to contribute to the full turbidity of the samples, even in the death stage of bacterial growth (Figure 3a), leading to erratic conclusions on bacterial growth estimation and MIC_50_ determination, as do samples that contribute to the overall turbidity of the suspensions, such as nanomaterials. On the contrary, luminescence measurements (i.e., LUX_490_) not only are not hampered by the turbidity of the suspensions, which can suffer fluctuations due to the presence of samples under analysis but are also thoroughly connected with the metabolism of the bacteria, allowing for a more reliable determination of the response of the bacteria to the tested compounds, and thus of their growth status at the time of the measurement. 

Unlike the other timestamps, after 24 h of incubation, bioluminescence intensity decreased exponentially, while OD_600_ measurements were maintained at higher values, since bacteria might be present in their stationary stage of growth. In this phase, no further growth occurred, stabilizing OD_600_ measurements. Furthermore, a decrease in metabolic activity was verified by the abrupt decrease in LUX_490_ intensity (Figure 3b). An estimation of bacterial growth using this data, after an incubation period of 24 h, might thus prove unreliable due to the reduced activity of the bacteria. The time growth curve of *E. coli* Lux without exposure to ofloxacin was thus evaluated to confirm the origin of such an intense decrease in LUX_490_. Whereas the OD_600_ profile through time followed an upward trend, reaching its maximum plateau at >24 h, the LUX_490_ profile only presented this uphill tendency for the first 8 h of the assay, falling abruptly after. This proves again the correlation between bioluminescence intensity and bacterial metabolic activity at this specific stage of bacterial growth, where both a higher metabolic activity and growth occur. Once again, at this stage of development, it is possible to estimate bacterial growth in response to stimuli through its tight relationship with metabolic activity [22]. 

As a last validation assay to the proposed approach, the new *E. coli* Lux has yet to be tested against the target of the work; that is, drug-delivery nanoparticles that can work as optical and colorimetric interferents in the evaluation of their own antimicrobial activity.

### 3.3. Nanoformulations Synthesis

**Figure 4:**
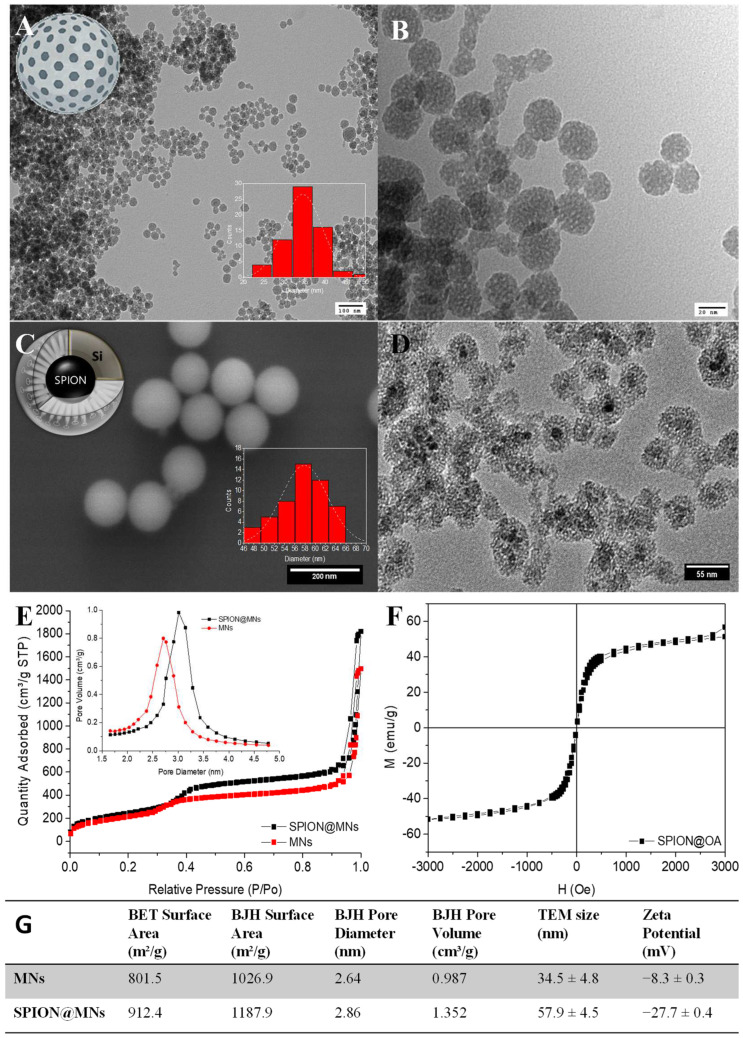
Physical characterization of MNs and SPION@MNs: MNs TEM micrographs confirming the particles’ mesoporosity, at 100 nm scale (**A**) and close up at 20 nm scale (**B**); inset: particle size distribution (*n* = 65). SPION@MNs SEM image, scale at 200 nm; inset: particle size distribution (*n* = 50) (**C**). TEM micrograph of SPION@MNs depicting single Fe_3_O_4_ magnetic cores encapsulated within silica mesoporous matrix, at 55 nm scale (**D**). The 72 K N_2_-isotherms of both MNs and SPION@MNs systems, again confirming their mesoporosity and availability for drug encapsulation (**E**). SPION@OA cores’ magnetization saturation curves (**F**). Summary of both MNs and SPION@MNs’ physical properties from surface area to pore diameter and volume, TEM size and surface zeta potential (**G**).

To describe a reliable and accurate bioluminescent antimicrobial activity assay, two distinct types of nanoparticles, loaded with a set of three drugs, were used as possible interferents to test the method’s robustness when compared to the performance of the commonly used OD-mediated approach. For that, MNs and SPION@MNs were successfully synthesized and obtained in the form of dry white and dark brown powders, respectively. This is evident in the spectroscopic analysis of the suspensions of the bare nanoparticles in water, with both having a significant wide light absorption, ranging from 650 to 200 nm (Figure S5). Both MNs and SPION@MNs showed a typical spherical conformation, with diameters of several tens of nanometers, and a mesoporous silica matrix, with pores of ca. 2.7 and 2.9 nm and available high surface areas of ca. 800 and 900 m^2^/g, respectively, typical of this type of mesoporous particles [11,38,39]. The slightly larger pore size of SPION@MNs owed it to the presence of the SPION stabilizer, oleic acid, that contributed to a certain degree of pore size modulation (Figure 4).

The successful incorporation of the magnetic oleic acid-coated iron-oxide nanocores within the mesoporous silica shell was not only confirmed by TEM imaging of SPION@MNs where clear core-shell SPION-MNs systems were present, but also by its magnetic susceptibility that arose from the magnetic core. Both types of particles showed the typical negative zeta potential (ZP), in water, of non-templated, bare mesoporous silica particles/shells that arise from their surface –OH groups. From their ZP results, we can predict the role of each nanoparticle as interferent in conventional OD assays, with more neutral ZPs like that of MNs leading to higher particle aggregation and higher interference with the OD signal and its reproducibility.

Following the synthesis of the nanomaterials, loading assays were conducted using epirubicin (EPI), doxorubicin (DOX), and ofloxacin (OFLO) as three model drugs (Figure S6), and both MNs and SPION@MNs were successfully loaded with either single-drug or 1:1 two-drugs formulations (i.e., DOX + OFLO or EPI + OFLO) (Table 1) This was also confirmed through the spectroscopic analysis of the loaded nanoparticles, where each drug absorbance profile overlapped with that of each nanoparticle (Figure S5). The loaded nanoparticles were also used as testing samples against the robustness of the luminescence-based assay. The particles’ capacity to release the loaded drugs was also assessed at pH = 7.4 and pH = 4.0, with both loaded MNs and SPION@MNs’ systems showing a significant release, similar to the previously reported MNs’ systems [38,39], and thus an additional potential antimicrobial activity (Table S1).

### 3.4. Nanoparticles’ Antimicrobial Activity against E. coli and E. coli Lux

The final assessment of the method’s robustness and usefulness was achieved by incubating *E. coli* Lux bacteria with the selected nanosystems (i.e., MNs and SPION@MNs-based nanoparticles), for a period of incubation of 8 h, previously determined as the optimal incubation period for bioluminescence measurements, with further determination of their MIC_50_ and/or MIC_90_. The assays were paralleled by incubating the same nanosystems against the parental *E. coli* strain, for 24 h, for comparison. According to what was stated in other sections, *E. coli* Lux and its parental *E. coli* strain were considered equivalents and thus equally susceptible to the selected antibiotic. While MNs’ systems were tested for concentrations of 362 to 0.5 µg/mL, via sequential dilutions, SPION@MNs’ systems were tested for 357 to 0.5 µg/mL. Each material was incubated without bacteria and used as negative controls and background signals for the calculation of bacterial growth. Similarly, bacteria were incubated in the same conditions, without the presence of any nanosystem, and taken as bacteria positive controls. The action of each free drug formulation was also assessed and used as drug positive controls (Figure S7).

**Figure 5:**
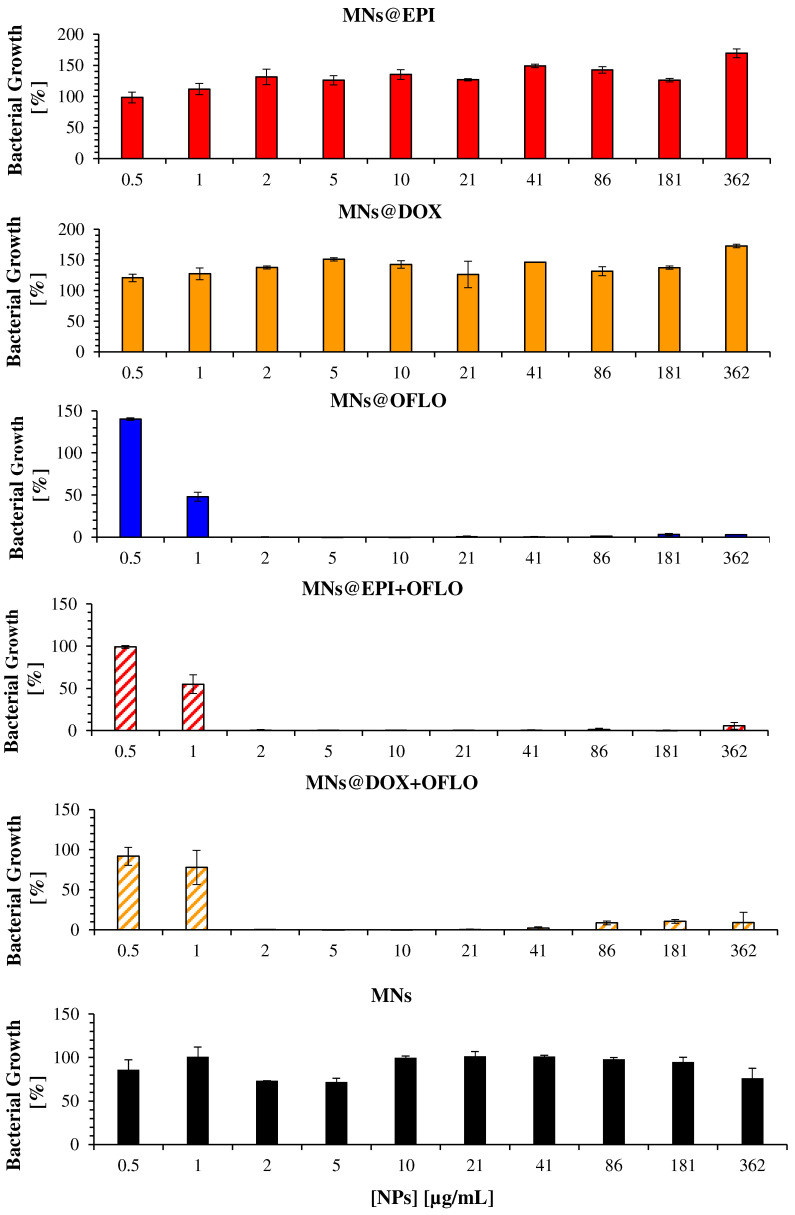
Antimicrobial activity of drug-loaded MNs on the parental *E. coli* strain, measured as the bacterial growth calculated by OD_600_, for 24 h assay.

**Figure 6:**
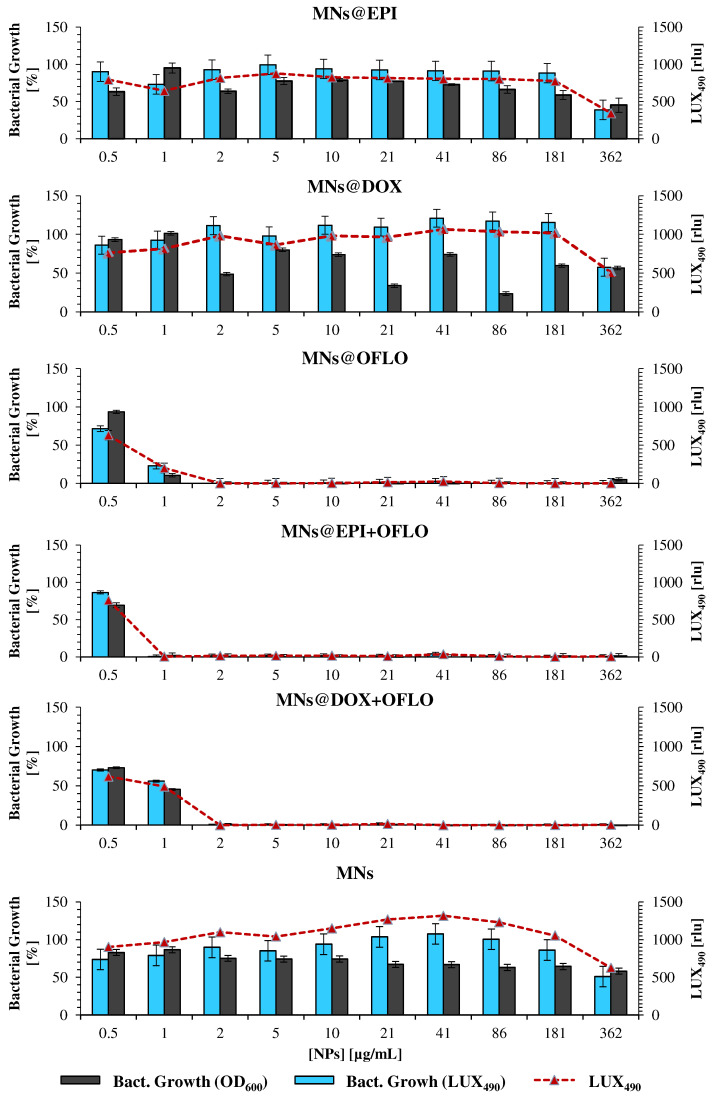
Antimicrobial activity of drug-loaded MNs on the mutant *E. coli* Lux strain, measured as bacterial growth calculated by OD_600_ and LUX_490_, for 8 h assay; raw LUX_490_ signal alone is depicted by the red dotted line and follows the same trend as the calculated bacterial growth.

From the incubation of MNs against both the parental *E. coli* strain (Figure 5) and the *E. coli* Lux transformant (Figure 6), it is clear that only those containing OFLO have significant inhibitory activity, as expected. Be it in single or dual-combinatory formulations (MNs@OFLO, MNs@EPI-OFLO, and MNs@DOX-OFLO), the obtained MIC_50_ was of ca. 1 µg/mL. Despite its undoubtful strong activity, the determination of a correct MIC_50_, via the OD_600_ signal after 24 h, was hampered by the visible large associated errors, as was the case with MNs@OFLO and MNs@DOX with errors of about ca. 50% and 20%, respectively. This issue persisted for the other MNs, whose associated errors accounted for a high variability (ca. 20%) of the calculated value (Figure 5). The calculated value was entirely dependent on the turbidity of the incubated suspension (OD_600_ signal), comprised of the turbidity of the bacteria and that of the nanomaterial, as well as on the latter’s homogeneity in the suspension, inducing the propagation of errors during MIC calculation and significant fluctuations in the obtained bacterial growth trend of *E. coli*. The vulnerability of OD_600_-based results to colorimetric samples (i.e., EPI and DOX) was perceived when looking at the obtained bacterial growth for each free drug formulation (Figure S7), with EPI and DOX having a fluctuating trend along the tested drug concentration gradient, despite their lower associated errors due to the lack of nanomaterials.

The introduction of the LUX_490_ signal as a probe for the calculation of bacterial growth, and consequently of each nanosystem MIC_50_, favored the elimination of high standard deviations and signal fluctuation, by discarding any interference caused by nanoparticles. The MIC_50_ obtained by the 8 h incubating *E. coli* Lux method, although similar for MNs@DOX-OFLO and MNs, differed from those obtained via 24 h OD_600_ for the rest of the systems. Whereas with the 24 h OD_600_ signal, no decrease in bacterial growth was registered for both 362 µg/mL MNs@EPI and MNs@DOX, with LUX_490_ growth, percentages relative to the control were of 38.73 ± 9.71% and 57.68 ± 8.26% for 362 µg/mL, respectively. While in the first approach, the results pointed towards a non-existence of a MIC, with the luminescence method, it was clear that the effect of both MNs@EPI and MNs@DOX at a concentration of 362 µg/mL was rather that of a MIC_50_, with a ca. 50% reduction of the metabolic activity, LUX_490_ signal, and consequently bacterial growth. A clear case of variability reduction was that of MNs@DOX + OFLO, for which the 8 h LUX_490_-calculated growth was 55.87 ± 7.62%, rather than the 77.64 ± 21.35% calculated from the 24 h OD_600_ signal, where no sensible determination of the MIC_50_ was possible. The same was obtained for MNs@EPI + OFLO, with the LUX_490_ signal pointing towards a growth of 0.39 ± 0.88% at a concentration of 1 µg/mL, and thus a possible MIC_90_ rather than an MIC_50_, as suggested by the results calculated from OD_600_.

**Figure 7:**
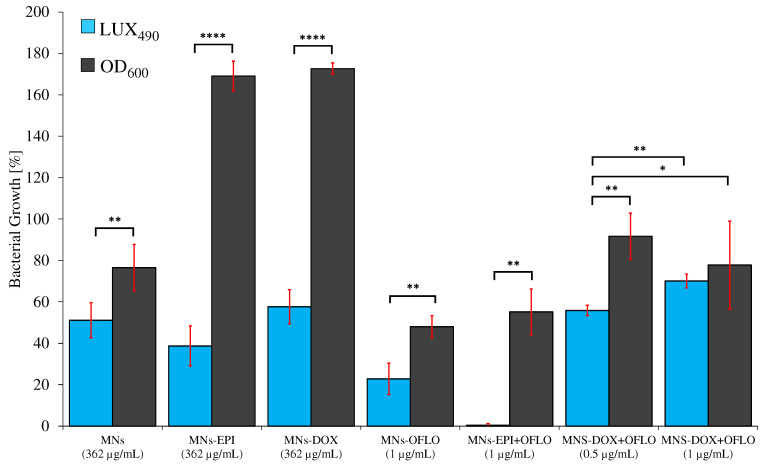
Statistical comparison of the obtained relative growth reduction, via OD_600_ 24 h-assaying the parental *E. coli* and LUX_490_ 8 h-assaying *E. coli* Lux, for all MNs’ systems (concentrations related to nanoparticle); statistically significant levels represented as * *p* ≤ 0.05, ** *p* ≤ 0.01, and **** ≡ *p* ≤ 0.0001.

The statistical analysis (*t*-test, α = 0.05, *n* = 4) of the differences between the obtained MIC_50_ and MIC_90_ from both methods (Table S2), and for each MN’s nanosystem, showed that, even though similar in some cases, the introduction of LUX_490_ gave significantly different growths from those obtained from OD_600_ (Figure 7 and Table S3). Thus, this justified a correction of the initially OD perceived MIC_50_ to the new LUX-related and metabolism-related MIC_50_.

**Figure 8:**
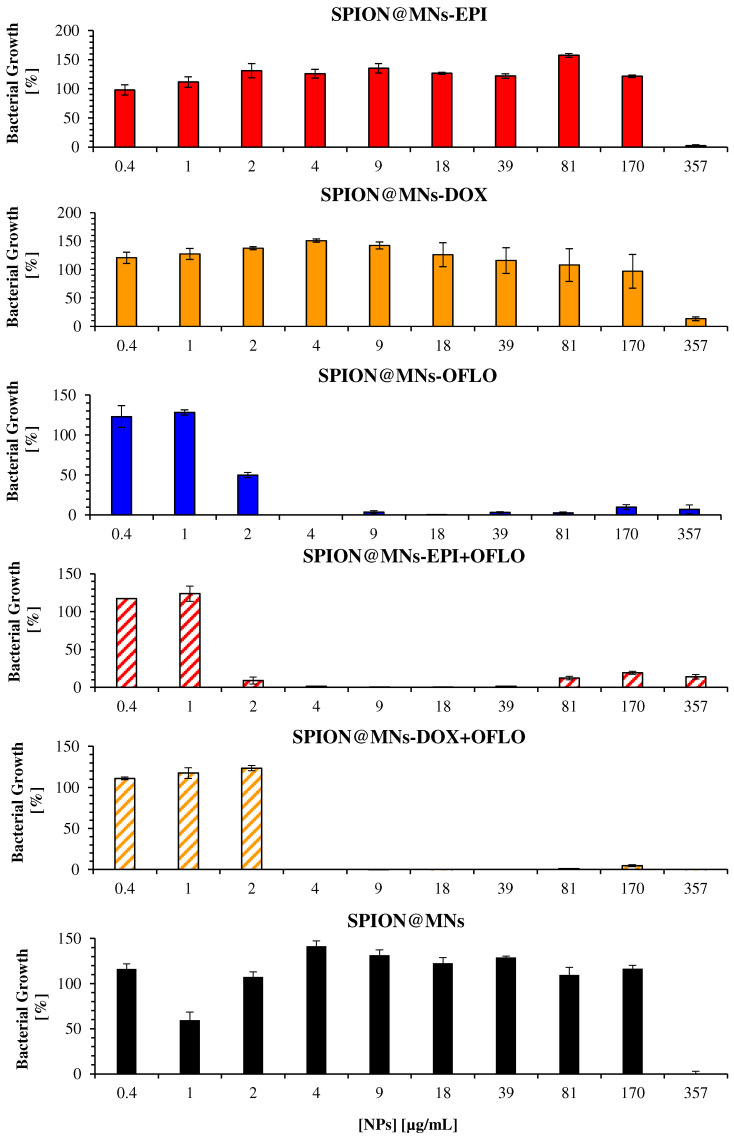
Antimicrobial activity of drug-loaded SPION@MNs on the parental *E. coli* strain, measured as the bacterial growth calculated by OD_600_, for 24 h assay.

**Figure 9:**
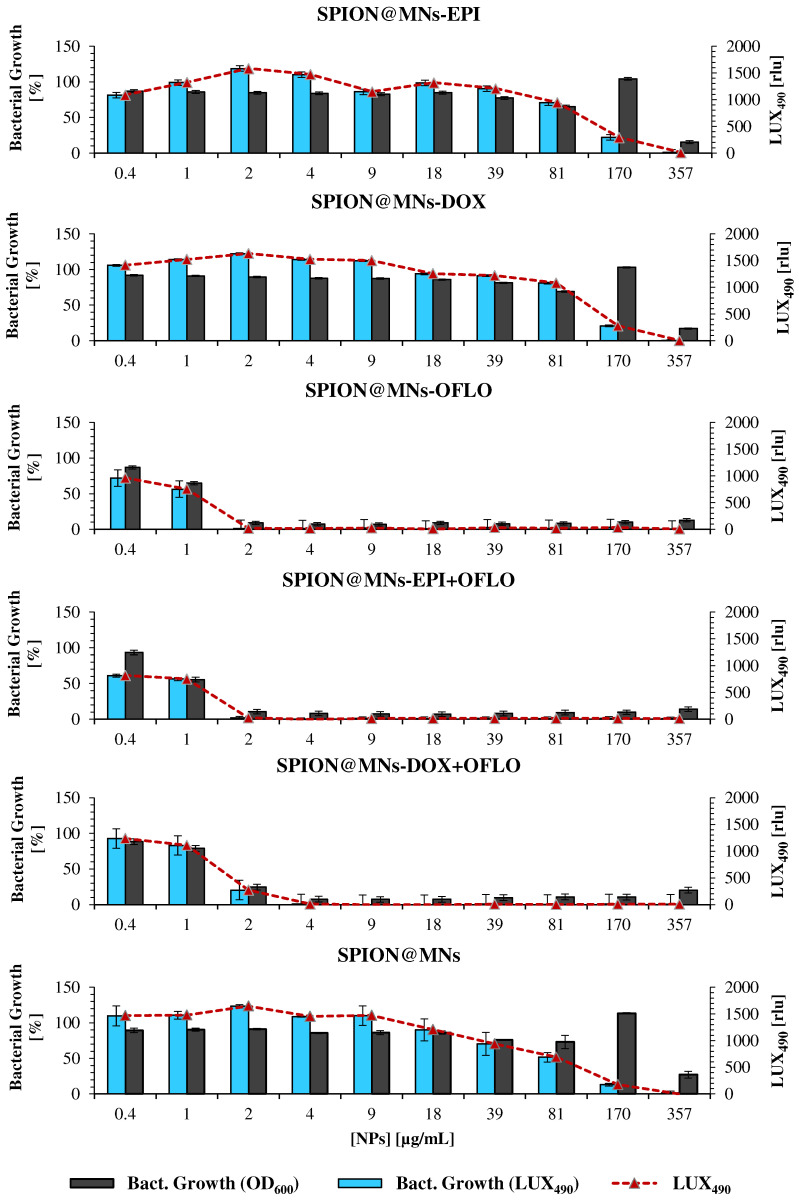
Antimicrobial activity of drug-loaded SPION@MNs on the mutant *E. coli* Lux strain, measured as bacterial growth calculated by OD_600_ and LUX_490_, for 8 h assay; raw LUX_490_ signal alone is depicted by the red dotted line and follows the same trend as the calculated bacterial growth.

A similar assessment was conducted for the SPION@MNs, by comparing the bacterial growth obtained from 24 h OD_600_ (Figure 8) and 8 h LUX_490_ (Figure 9) signals. Regarding SPION@MNs, SPION@MNs-EPI, and SPION@MNs-DOX nanosystems, whereas the 24 h OD_600_-calculated MIC_50_ stayed between a concentration range of 357–170 µg/mL, a shift in the obtained MICs to smaller concentrations of 170 to 81 µg/mL was seen when the 8 h LUX_490_ signal was used. In this last case, a clear decreasing growth gradient was seen as the particle concentration increased, without the abrupt decreases that were registered for 24 h OD_600_-calculated growth, as well as for 8 h OD_600_ growth in *E. coli* Lux. The possible MIC_90_ of these nanosystems, however, was the same, with a growth of ca. 0% for a concentration of 357 µg/mL, calculated for both LUX_490_ and OD_600_. In brief, by using the LUX_490_ signal after 8 h of incubation, fluctuations in the growth gradient can be eliminated and a more sensible determination and distinction of MIC_50_ and MIC_90_ is possible.

When comparing the results obtained, through both methods, for SPION@MNs-OFLO and SPION@MNs-EPI + OFLO nanosystems, the same trend was seen with 8 h LUX_490_-calculated growth that produced a smoother decreasing gradient, with less variability, as the particle concentration increased. In these cases, a correct determination of the MIC_50_ was possible, with SPION@MNs-OFLO and SPION@MNs-EPI + OFLO having significant inhibitory activity at concentrations of 1 and 0.4 µg/mL, respectively, and a common MIC_90_ at 2 µg/mL. Lastly, a more sensible determination of both MIC_50_ and MIC_90_ of SPION@MNs-DOX + OFLO nanoparticles was also possible, with an 8 h LUX_490_ signal pointing to a 50% inhibitory concentration at 2 µg/mL, contrary to those obtained from the 24 h OD_600_ signal, and a common 90% inhibitory concentration at 4 µg/mL.

The substitution of the OD_600_ signal for that of LUX_490_ as a more sensible probe for bacterial growth calculation, and the correct determination of MIC_50_ and MIC_90_, was again supported by the statistical analysis of growth differences between the obtained MIC_50_ and MIC_90_ for both methods (Tables S4 and S5). As can be seen in Figure S8, the obtained growth from LUX_490_ not only was significantly different (for α = 0.05, *n* = 4) between adjacent concentrations, but also between those obtained from the 24 h OD_600_ signal.

Lastly, a test to the new method’s reproducibility was achieved by repeatedly assaying *E. coli* Lux with all OFLO-containing drug combinations, in different days, and with different operators. The statistical analysis between the obtained MIC_50_ and MIC_90_ of both assays (2-tailed *t*-test to the means, for α = 0.05, *n* = 2) confirmed that there was no significant statistical difference between the results of each assay, and thus that the method is reproducible (Figure S9).

## 4. Conclusions

The herein detailed luminescence-based antimicrobial activity assay presents several advantages when compared to the traditional OD_600_-based assays. The first main advantage is the reduction of incubation time from 24 to only 8 h. This is related to the time at which optimal luminescence is obtained after incubation and at which bacterial growth is in its exponential phase. This shorter incubation time allows for the acquisition of results concerning bacterial growth in the same day the assay is conducted. 

The second main advantage relates to the type of measurements. Unlike OD_600_-based assays, by measuring LUX_490_ it is possible to not only calculate definite percentages of bacterial growth, but also to determine and more easily distinguish between MIC_50_ and MIC_90_ concentrations by the direct analysis of the LUX_490_ raw data. Moreover, it allows for a reduction in the overall variability and uncertainty of the obtained results. This is of extreme importance when working with nanoparticle suspensions and colorimetric compounds, whose turbidity and color (usual interferents in OD_600_ calculations) no longer need to be taken into consideration in the overall MIC calculation. Such is the case of MNs and SPION@MNs’ colorimetric formulations (i.e., with DOX and EPI), where otherwise determined MIC_50_ were correctly assessed as rather MIC_90_, moving the real assessment of MIC50 to lower concentrations. This is possible since luminescence is directly correlated with the metabolic response of the bacteria to the provided stimulus, and not to the physical behavior of the suspension, providing more accurate results.

In light of current worldwide efforts to find standard and reliable toxicological procedures for the determination of nanoparticles’ toxicity and antimicrobial activities, we believe that this approach is an important tool that contributes to the standardization of methods in potential industrial applications of nanoparticles, as it reduces samples’ background interference and focuses on luminescence as a direct probe for bacterial metabolic activity and growth, as well as the correct determination of the antimicrobial activity of said nanomaterials.

## Figures and Tables

**Table 1:** MNs and SPION@MNs’ loading assay loading percentages and encapsulation efficiency (EE) for single and dual-combinatory formulations of EPI, DOX, and OFLO.

Nanomaterial	Drug		Loading %	EE (mg/g)
MNs	EPI		87.1 ± 9.6	21.0 ± 3.9
	DOX		72.4 ± 4.7	19.1 ± 0.1
	OFLO		38.8 ± 12.9	9.8 ± 3.0
	EPI + OFLO	*Epirubicin*	90.9 ± 6.7	22.6 ± 0.9
		*Ofloxacin*	58.1 ± 8.3	14.4 ± 1.6
	DOX + OFLO	*Doxorubicin*	83.7 ± 14.1	21.3 ± 0.6
		*Ofloxacin*	57.8 ± 21.2	14.5 ± 3.3
SPION@MNs	EPI		100.0 ± 0.0	25.0 ± 0.0
	DOX		100.0 ± 0.0	25.0 ± 0.0
	OFLO		99.7 ± 0.2	24.9 ± 0.1
	EPI + OFLO	*Epirubicin*	88.3 ± 9.1	22.1 ± 2.3
		*Ofloxacin*	47.3 ± 8.4	11.8 ± 2.1
	DOX + OFLO	*Doxorubicin*	80.5 ± 18.7	20.1 ± 4.7
		*Ofloxacin*	47.2 ± 10.0	11.8 ± 2.5

## Data Availability

Not applicable.

## Supplementary Materials

The following supporting information can be downloaded at: https://www.mdpi.com/article/10.3390/nano12132164/s1. Table S1: Loaded MNs and SPION@MNs cumulative release assay results, in PBS 0.01 M pH 7.4 and pH 4.0, at 24 h.; Table S2: Inhibitory concentrations of all SPION@MNs systems against *E. coli* and *E. coli* Lux, for 24 h and 8 h assays, with respective bacterial growth; Table S3: Statistical analysis of the bacterial growth averages obtained from LUX490 (8 h) and OD600 (24 h), for all inhibitory concentrations of MNs systems against *E. coli* Lux and *E. coli*, respectively; Table S4: Inhibitory concentrations of all SPION@MNs systems against *E. coli* and *E. coli* Lux, for 24 h and 8 h assays, with respective bacterial growth; Table S5: Statistical analysis of the bacterial growth averages obtained from LUX490 (8 h) and OD600 (24 h), for all inhibitory concentrations of SPION@MNs systems against *E. coli* Lux and *E. coli*, respectively; Figure S1: pMV306G13 + Lux plasmid constitution; Figure S2: Plates with the dilutions 10^−4^ (TSA Lux3), 10^−5^ (TSA Lux4) and 10^−6^ CFU (TSA Lux5) for CFU counting of luminescent *E. coli* Lux suspension; Figure S3: The susceptibility of the wild-type parent *E. coli* (ATCC® 8739TM) to ofloxacin, given as raw OD600 vs. concentration of OFLO; Figure S4: *E. coli* Lux growth data under shaking (a,b) and static (d,e) conditions; Figure S5: Absorbance spectra of MNs and SPION@MNs, in water; Figure S6: Structural representation of all model drugs and respective absorbance spectra: epirubicin (EPI), doxorubicin (DOX) and ofloxacin (OFLO); Figure S7: Antimicrobial activity of single and combinatory drug controls on the parental *E. coli* strain; Figure S8: Statistical comparison of the obtained relative growth reduction; Figure S9: Statistical comparison of the MIC50/MIC90 obtained, via LUX490 8 h-assaying of *E. coli* Lux, for trial 1 (t1) and trial 2 (t2), of all OFLO-containing drug combinations. References [28,52,53] are cited in the supplementary materials.
