# Supplementary material for: Validation of a Standard Luminescence Method for the Fast Determination of the Antimicrobial Activity of Nanoparticles in Escherichia coli
Source: Nanomaterials (Basel). 2022 Jun 23;12(13):2164. doi: 10.3390/nano12132164 (PMC9268724; doi:10.3390/nano12132164)
Supplement: Supplementary file 1 [file nanomaterials-12-02164-s001.zip › nanomaterials-1774120-supplementary.pdf]

Supplementary Materials

Validation of a Standard Luminescence Method for the Fast Determination of the Antimicrobial Activity of Nanoparticles in *Escherichia Coli*

Gonçalo A. Marcelo [1], Joana Galhano [1], Maria Paula Duarte [2,*], José Luis Capelo-Martínez [1,3], Carlos Lodeiro [1,3] and Elisabete Oliveira [1,3,*]

[1] BIOSCOPE Group, LAQV-REQUIMTE, Chemistry Department, NOVA School of Science and Technology, FCT NOVA, NOVA University Lisbon, 2829-516 Caparica, Portugal; goncalo.marcelo9@gmail.com (G.A.M.); j.galhano@campus.fct.unl.pt (J.G.); jlcm@fct.unl.pt (J.L.C.-M.); cle@fct.unl.pt (C.L.)
[2] MEtRICs, NOVA School of Science and Technology, NOVA University Lisbon, 2829-516 Caparica, Portugal
[3] PROTEOMASS Scientific Society, Rua dos Inventores, Madam Parque, Caparica Campus, 2825-182 Caparica, Portugal
* Correspondence: mpcd@fct.unl.pt (M.P.D.); ej.oliveira@fct.unl.pt (E.O.)

1. Experimental Procedures

1.1. Plasmid Extraction and Purification

For the extraction of the pMV306G13+Lux plasmid [1], the acquired plasmid-containing growth strain, *E. coli* DH5α, was incubated in TSB for 16 h, at 37 °C, 210 rpm, and the plasmid was extracted by applying the protocol detailed in the Thermo Scientific™ GeneJET Plasmid Miniprep Kit #K0502, yielding a concentration of 27 ng/mL.

Subsequently, the pDNA solution was de-salted to remove salts that could interfere with the electroporation procedure. Briefly, to precipitate the pDNA, 5 µL of a 3 M sodium acetate solution (pH 5.2) and 100 µL of ethanol 96% were added to 50 µL of the plasmid solution. The mixture was left at −70 °C for 15 minutes and then, centrifuged (12,000× *g*, 15 min) at 4 °C. The pellet was later washed with ethanol 70% and left to dry at 37 °C. Finally, the dry pellet was resuspended in 50 µL of sterile deionized (DI) Milli-Q water and stored at −70 °C. The resulting plasmid solution was quantified in a Nanodrop 1000 spectrophotometer, in Nucleic Acids mode. Purity was accessed according to the 260/280 nm ratio.

1.2. Development of the new E. coli Lux strain

1.2.1. Competence induction

Competent bacteria were obtained following an adapted protocol from the MicroPulser™ Electroporation Apparatus Operating Instructions and Applications Guide from BioRad126 [2]. In detail, *Escherichia coli* ATCC® 8739™ was grown in L-broth (10 g tryptone, 5 g yeast extract and 5 g NaCl in 1 L Milli-Q water) overnight, at 37 °C and 210 rpm. Then, 500 mL of L-broth were inoculated with 1/100 volume of the bacterial culture. The suspensions were incubated at 37 °C and 250 rpm, until an OD 600 nm of 0.5–0.7 was achieved. The cells were cooled on ice for 20 min and centrifuged (4000× *g*, 15 min) at 4 °C. All subsequent centrifugations were conducted with the same parameters. Supernatants were discarded and the pellet resuspended in 500 mL of cold 10% glycerol, followed by another centrifugation cycle. This step was repeated 2 additional times, decreasing the resuspending volume of 10% glycerol to 250 and 20 mL, respectively. Following this centrifugation step, the supernatant was again discarded, and the pellet resuspended in 2 mL of cold 10% glycerol. From this suspension, 150 µL aliquots were prepared and stored at −70 °C.

1.2.2. Bacterial Transformation by Electroporation

Electroporation protocols were performed in the Laboratory of Genetics of NOVA Medical School Centro de Estudos de Doenças Crónicas (CEDOC). 8 μL of the purified pMV306G13 + Lux plasmid was added to a 150 μL competent bacterial aliquot, followed by a gentle homogenization. The suspension was incubated for a brief period on ice and was then transferred to a cold electroporation cuvette. The electroporation parameters were of 2.1 kV, 100 Ω and 25 μF. After the pulse, a τ = 2.4 was obtained.

The sample was retrieved, and 1 mL of SOC medium (2% tryptone, 0.5% yeast extract, 10 mM NaCl, 2.5 mM KCl, 10 mM $MgCl_2$, 10 mM $MgSO_4$ and 20 mM monohydrated glucose) was added. Bacterial suspensions were incubated at 37 °C and 210 rpm, for *ca.* 1 h, and then plated in kanamycin-TSA (35 μg/mL kanamycin).

Plates were incubated at 37 °C, overnight. Transformed *E. coli* cells (*E. coli* Lux strain) were streaked in TSA plates, supplemented with 35 μg/mL of kanamycin, and incubated at 37 °C overnight. Finally, isolated luminescent colonies were inoculated into TSB (35 μg/mL) and incubated at 37 ºC and 210 rpm. Glycerol stocks (15%) were prepared and stored at −70 °C.

2. Tables

Table S1. Loaded MNs and SPION@MNs cumulative release assay results, in PBS 0.01M pH 7.4 and pH 4.0, at 24h.

**Nanomaterial**	**Dr g**		**Release % (pH 7.4)**	**Release % (pH 4.0)**
MNs	EPI		12.7 ± 3.2	45.8 ± 2.2
	DOX		8.4 ± 0.2	15.3 ± 0.02
	OFLO		41.5 ± 1.5	52.1 ± 1.3
	EPI + OFLO (EO)	*Epirubicin*	12.7 ± 0.7	57.3 ± 0.5
		Ofloxacin	46.7 ± 2.2	67.4 ± 0.8
	DOX + OFLO (DO)	*Doxorubicin*	25.0 ± 0.3	62.1 ± 4.3
		Ofloxacin	38.9 ± 1.4	43.5 ± 2.3
SPION@MNs	EPI		8.3 ± 2.0	100.0 ± 57.0
	DOX		10.7 ± 4.3	44.6 ± 23.9
	OFLO		23.7 ± 0.9	20.8 ± 0.5
	EPI + OFLO (EO)	*Epirubicin*	14.5 ± 1.3	80.7 ± 39.3
		Ofloxacin	38.4 ± 9.8	43.3 ± 7.6
	DOX + OFLO (DO)	*Doxorubicin*	23.1 ± 9.4	63.0 ± 21.6
		Ofloxacin	28.8 ± 6.4	58.7 ± 10.4

Table S2. Inhibitory concentrations of all SPION@MNs systems against *E. coli* and *E. coli* Lux, for 24 h and 8 h assays, with respective bacterial growth (average ± standard deviation).

**Nanosystem**	**[NP] [µg/mL]**	**LUX_{490}** Bacterial growth [%]		**OD_{600}** Bacterial growth [%]	
		Average (n = 4)	Standard deviation	Average (n = 4)	Standard deviation
MNs	362	51.11	±8.5	76.51	±11.32
MNs-EPI	362	38.73	±9.71	169.13	±7.19
MNs-DOX	362	57.68	±8.26	172.68	±2.73
MNs-OFLO	1	22.85	±7.62	48.01	±5.38
MNs-EPI + OFLO	1	0.39	±0.88	55.20	±11.10
MNS-DOX + OFLO	0.5	55.87	±2.49	77.80	±21.30
MNS-DOX + OFLO	1	70.10	±3.37	91.70	±11.20

Table S3. Statistical analysis of the bacterial growth averages obtained from LUX_{490} (8 h) and OD_{600} (24 h), for all inhibitory concentrations of MNs systems against *E. coli* Lux and *E.coli*, respectively; *t*-test (two-tailed) for $n = 4$, $\alpha = 0.05$, $t\ (3, 0.05/2) = 3.128$ (H_0: $\overline{X}_1 = \overline{X}_2$, H_1: $\overline{X}_1 \neq \overline{X}_2$, where 1 and 2 stand for LUX or OD depending on the type of interaction).

**Nanosystem**	**[NP] interaction [µg/mL]**	***t*-score**	**Result**	***p*-value**	**Classification**	**Type of interaction**
MNs	362 × 362	−6.80	Reject H_0	0.00650	**	LUX × OD
MNs-EPI	362 × 362	−39.97	Reject H_0	0.00003	****	LUX × OD
MNs-DOX	362 × 362	−29.49	Reject H_0	0.00009	****	LUX × OD
MNs-OFLO	1 × 1	−9.32	Reject H_0	0.00261	**	LUX × OD
MNs-EPI + OFLO	1 × 1	−9.91	Reject H_0	0.00219	**	LUX × OD
MNS-DOX + OFLO	0.5 × 0.5	−6.56	Reject H_0	0.00720	**	LUX × OD
	1 × 1	−0.73	Accept H_0	0.51823	ns	LUX × OD
	0.5 × 1	−4.04	Reject H_0	0.02722	*	LUX × OD
	0.5 × 1	−2.07	Accept H_0	0.12987	ns	OD × LUX
	0.5 × 1	−12.52	Reject H_0	0.00109	**	LUX × LUX
	0.5 × 1	0.02	Accept H_0	0.98529	ns	OD × OD

ns ≡ p > 0.05, * ≡ p ≤ 0.05, ** ≡ p ≤ 0.01, ***** ≡ p ≤ 0.0001

Table S4. Inhibitory concentrations of all SPION@MNs systems against *E. coli* and *E. coli* Lux, for 24 h and 8 h assays, with respective bacterial growth (average ± standard deviation).

Nanosystem	[NP] [μg/mL]	LUX_{490}		OD_{600}	
		Bacterial growth [%]		Bacterial growth [%]	
		Average (n = 4)	Standard deviation	Average (n = 4)	Standard deviation
SPION@MNs	81	51.60	±6.66	109.52	±8.33
SPION@MNs	357	1.83	±2.28	−4.81	±7.69
SPION@MNs-EPI	170	21.91	±0.95	121.48	±1.99
SPION@MNs-EPI	357	0.93	±1.11	2.37	±1.77
SPION@MNs-DOX	170	20.86	±2.27	96.93	±29.68
SPION@MNs-DOX	357	0.40	±1.85	13.27	±3.28
SPION@MNs-OFLO	1	56.28	±3.81	128.00	±3.31
SPION@MNs-OFLO	2	1.30	±0.58	49.90	±3.30
SPION@MNs-EPI + OFLO	1	56.24	±6.135	123.72	±10.03
SPION@MNs-EPI + OFLO	2	1.94	±0.85	9.06	±4.71
SPION@MNS-DOX + OFLO	1	83.01	±1.79	117.47	±6.49
SPION@MNS-DOX + OFLO	2	20.30	±7.25	123.56	±3.14
SPION@MNS-DOX + OFLO	4	0.78	±1.22	0.21	±0.05

Table S5. Statistical analysis of the bacterial growth averages obtained from LUX_{490} (8 h) and OD_{600} (24 h), for all inhibitory concentrations of SPION@MNs systems against *E.coli* Lux and *E.coli*, respectively; *t*-test (two-tailed) for $n = 4$, $\alpha = 0.05$, $t\ (3, 0.05/2) = 3.128$ (H_0: $\overline{X}_1 = \overline{X}_2$, H_1: $\overline{X}_1 \neq \overline{X}_2$, where 1 and 2 stand for LUX or OD depending on the type of interaction).

Nanosystem	[NP] interaction [μg/mL]	*t*-score	Result	*p*-value	Classification	Type of interaction
SPION@MNs	81 × 81	−23.18	Reject H_0	0.00018	***	LUX × OD
	357 × 357	1.81	Accept H_0	0.16858	ns	LUX × OD
	81 × 357	29.34	Reject H_0	0.00009	****	LUX × OD
	81 × 357	−26.89	Reject H_0	0.00011	***	OD × LUX
	81 × 357	15.90	Reject H_0	0.00054	***	LUX × LUX
	81 × 357	71.66	Reject H_0	< 0.00001	****	OD × OD
SPION@MNs EPI	170 × 170	−113.81	Reject H_0	< 0.00001	****	LUX × OD
	357 × 357	−2.09	Accept H_0	0.12753	ns	LUX × OD
	170 × 357	26.21	Reject H_0	0.00012	***	LUX × OD
	170 × 357	−145.79	Reject H_0	< 0.00001	****	OD × LUX
	170 × 357	73.35	Reject H_0	< 0.00001	****	LUX × LUX
	170 × 357	259.98	Reject H_0	< 0.00001	****	OD × OD
SPION@MNs DOX	170 × 170	−5.14	Reject H0	0.01426	*	LUX × OD
	357 × 357	−9.50	Reject H_0	0.00247	**	LUX × OD
	170 × 357	6.42	Reject H_0	0.00766	**	LUX × OD
	170 × 357	−6.52	Reject H_0	0.00734	**	OD × LUX
	170 × 357	30.97	Reject H_0	0.00007	****	LUX × LUX
	170 × 357	5.67	Reject H0	0.01085	*	OD × OD
SPION@MNs OFLO	1 × 1	−76.20	Reject H_0	0.00334	**	LUX × OD
	2 × 2	−29.92	Reject H_0	0.00008	****	LUX × OD
	1 × 2	6.71	Reject H_0	0.00675	**	LUX × OD
	1 × 2	−77.77	Reject H_0	0.00083	***	OD × LUX
	1 × 2	29.22	Reject H_0	0.00009	****	LUX × LUX
	1 × 2	607.55	Reject H_0	< 0.00001	****	OD × OD
SPION@MNs EPI-OFLO	1 × 1	−17.01	Reject H_0	0.00044	***	LUX × OD
	2 × 2	−3.08	Accept H_0	0.05426	ns	LUX × OD
	1 × 2	23.97	Reject H_0	0.00016	***	LUX × OD
	1 × 2	−24.37	Reject H_0	0.00015	***	OD x LUX
	1 × 2	17.87	Reject H_0	0.00038	***	LUX × LUX
	1 × 2	25.89	Reject H_0	0.00013	***	OD × OD
SPION@MNs DOX + OFLO	1 × 1	−11.05	Reject H_0	0.00159	**	LUX × OD
	2 × 2	−31.63	Reject H_0	0.00007	****	LUX × OD
	4 × 4	0.94	Accept H_0	0.41643	ns	LUX × OD
	1 × 2	−31.46	Reject H_0	0.00007	****	LUX × OD
	1 × 4	92.14	Reject H_0	< 0.00001	****	LUX × OD
	2 × 4	5.55	Reject H_0	0.01156	*	LUX × OD
	1 × 2	60.38	Reject H_0	0.00001	****	OD × LUX
	1 × 4	36.60	Reject H_0	0.00004	****	OD × LUX
	2 × 4	84.74	Reject H_0	< 0.00001	****	OD × LUX
	1 × 2	17.87	Reject H_0	0.00038	***	LUX × LUX
	1 × 4	124.20	Reject H_0	< 0.00001	****	LUX × LUX

	2 × 4	5.47	Reject H_0	0.01203	*	LUX × LUX
	1 × 2	−2.14	Accept H_0	0.12162	ns	OD × OD
	1 × 4	36.13	Reject H_0	0.00005	****	OD × OD
	2 × 4	78.51	Reject H_0	< 0.00001	****	OD × OD

ns ≡ p > 0.05, * ≡ p ≤ 0.05, ** ≡ p ≤ 0.01, *** ≡ p ≤ 0.001, ***** ≡ p ≤ 0.0001

3. Figures

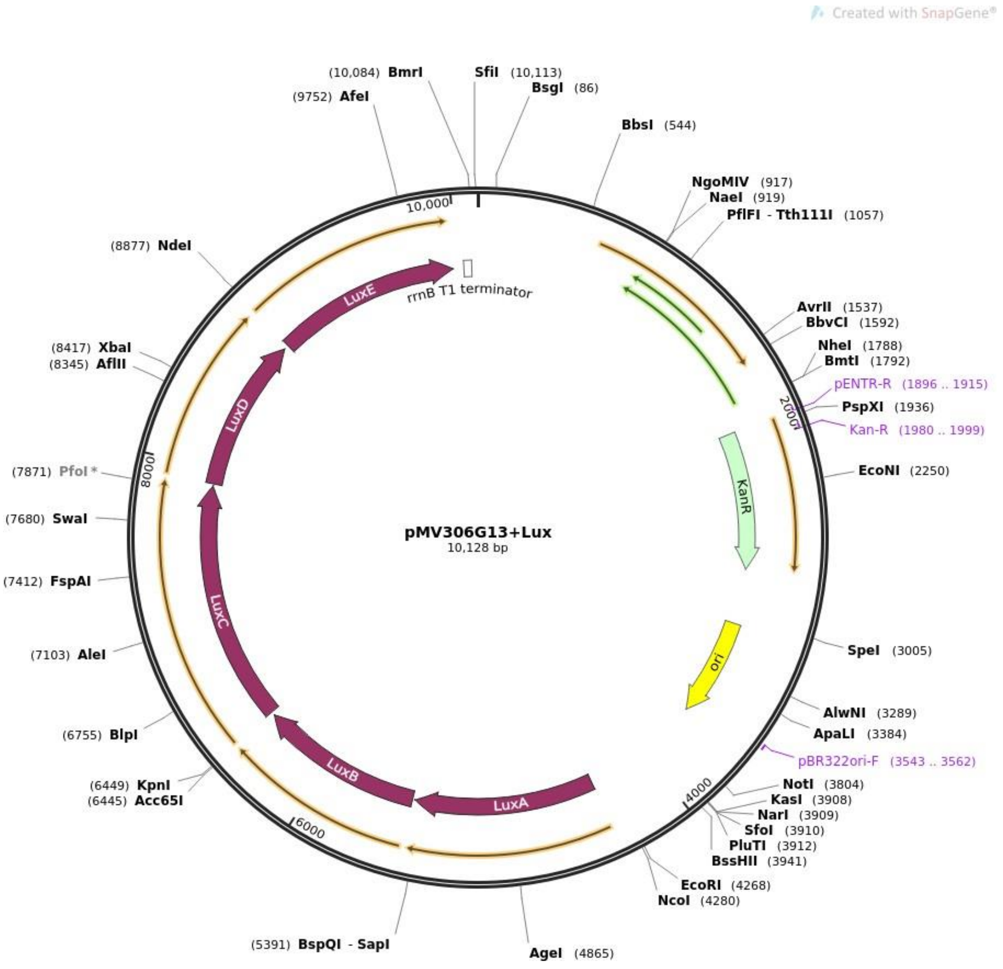

Figure S1. pMV306G13 + Lux plasmid constitution. Reproduced from [3].

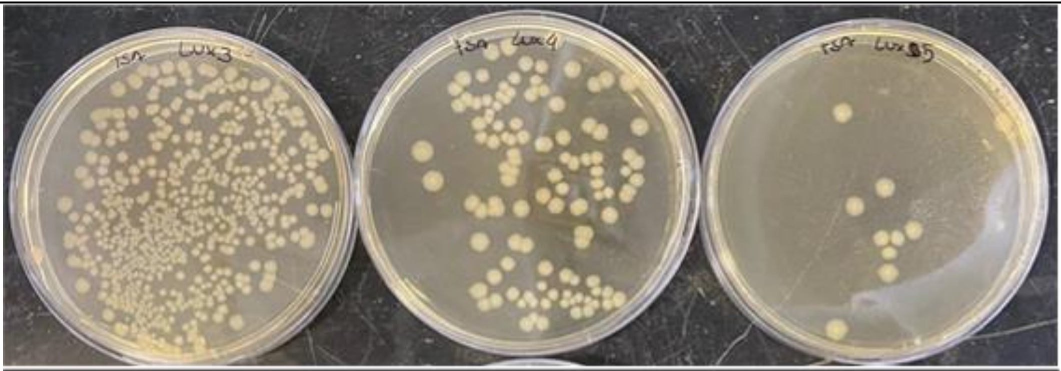

Plate	Expected CFUs	Counted CFUs
TSA Lux3 (100 μL of 10^{-4} dilution)	1000	>300
TSA Lux4 (100 μL of 10^{-5} dilution)	100	115
TSA Lux5 (100 μL of 10^{-6} dilution)	10	10

Figure S2. Plates with the dilutions 10^{-4} (TSA Lux3), 10^{-5} (TSA Lux4) and 10^{-6} CFU (TSA Lux5) for CFU counting of luminescent *E. coli* Lux suspension.

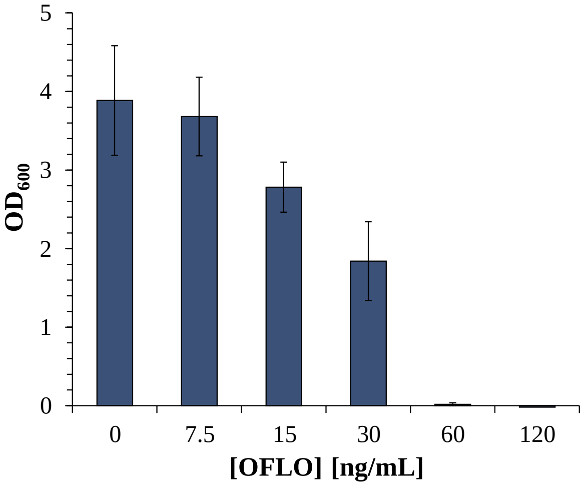

Figure S3. The susceptibility of the wild-type parent E.coli (ATCC® 8739™) to ofloxacin, given as raw OD_{600} vs. concentration of OFLO.

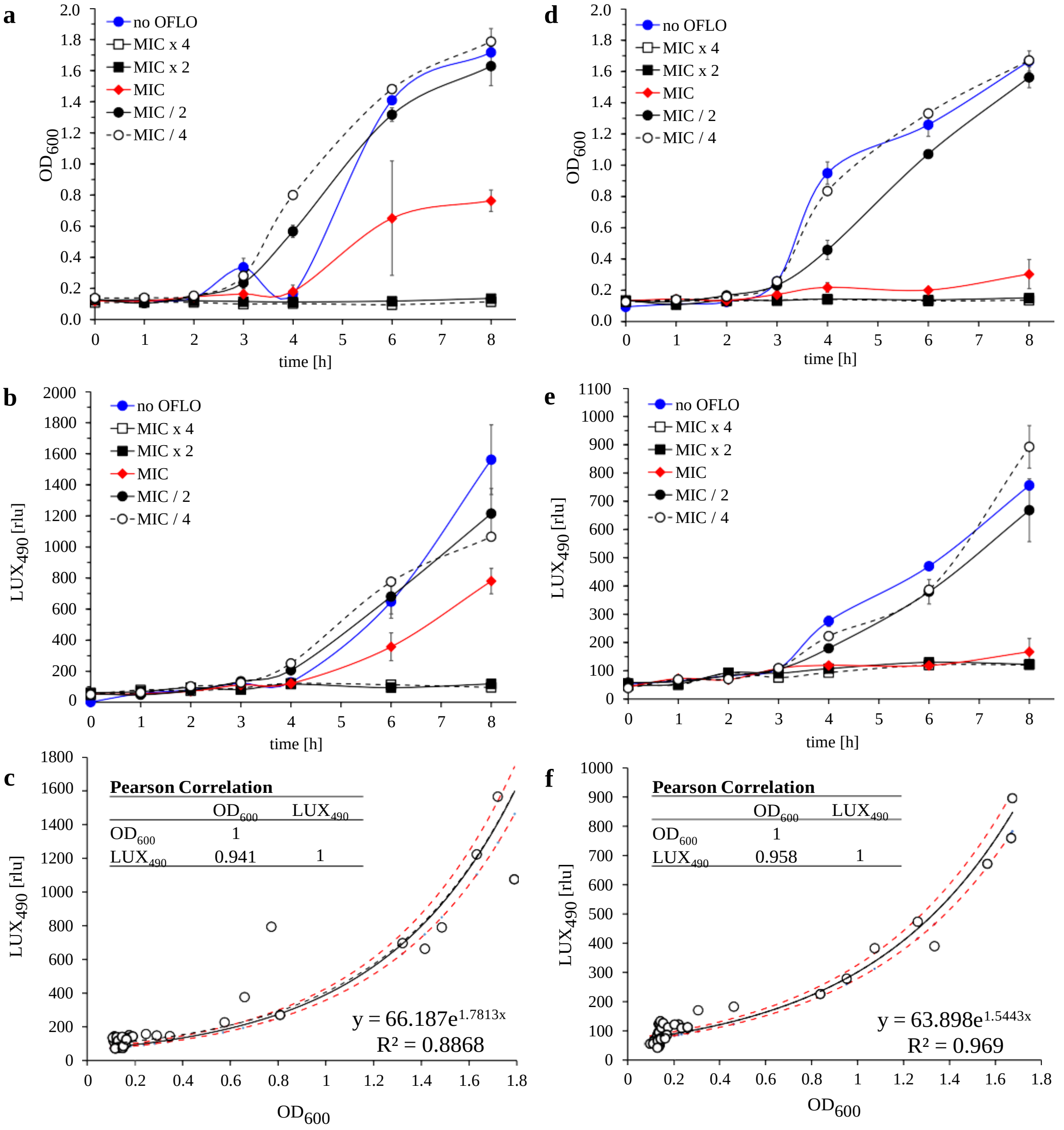

Figure S4. E.coli Lux growth data under shaking (**a**,**b**) and static (**d**,**e**) conditions, collected as OD_{600} and LUX_{490}; successive dilutions from MIC = 30 ng/mL. Plotting of all average OD_{600} vs. LUX_{490} with respective exponential regressions and Pearson correlations, for shaking (**c**) and static (**f**) conditions.

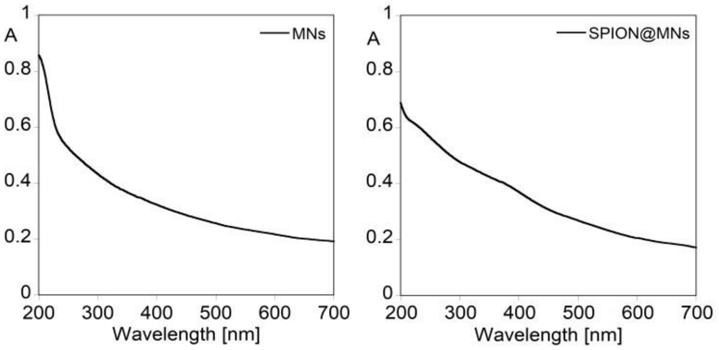

Figure S5. Absorbance spectra of MNs and SPION@MNs, in water.

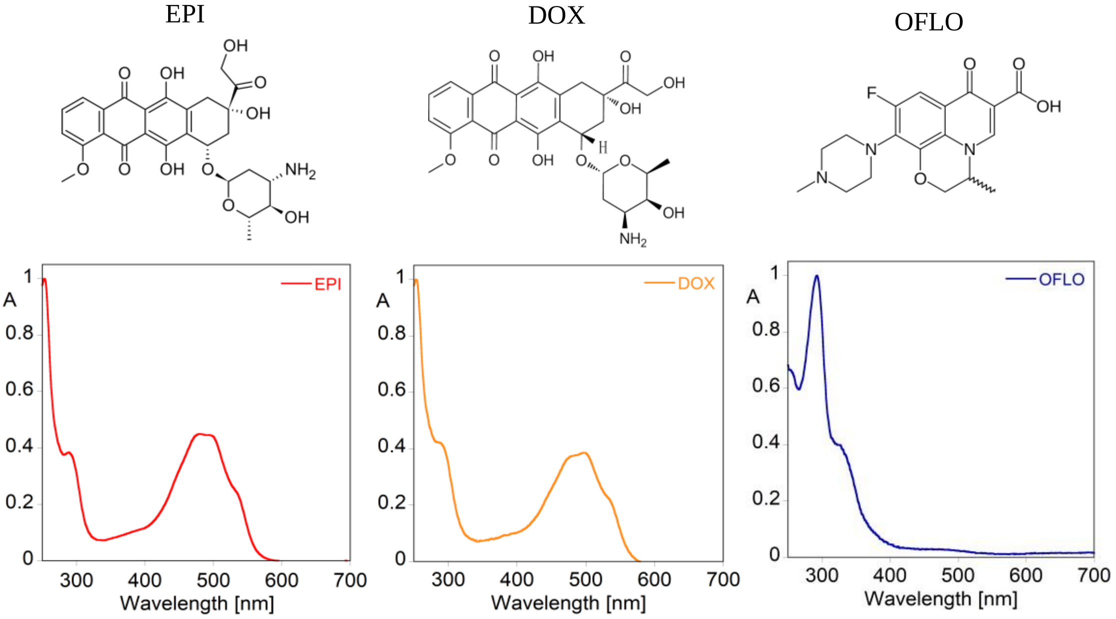

Figure S6. Structural representation of all model drugs and respective absorbance spectra: epirubicin (EPI), doxorubicin (DOX) and ofloxacin (OFLO).

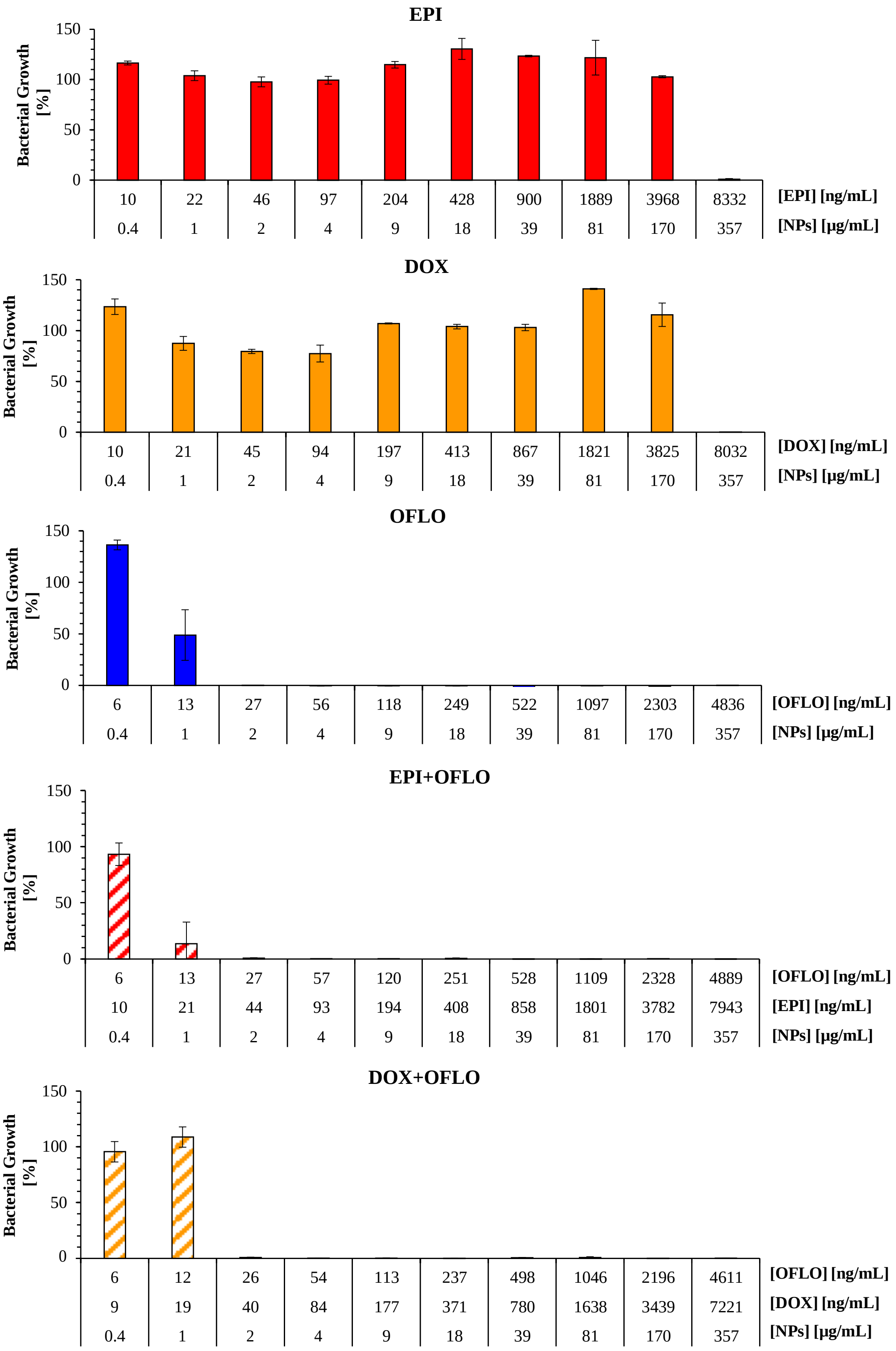

Figure S7. Antimicrobial activity of single and combinatory drug controls on the parental *E. coli* strain, measured as the bacterial growth calculated by OD_{600}; drug concentrations in ng/mL and respective equivalent nanoparticles concentrations in µg/mL.

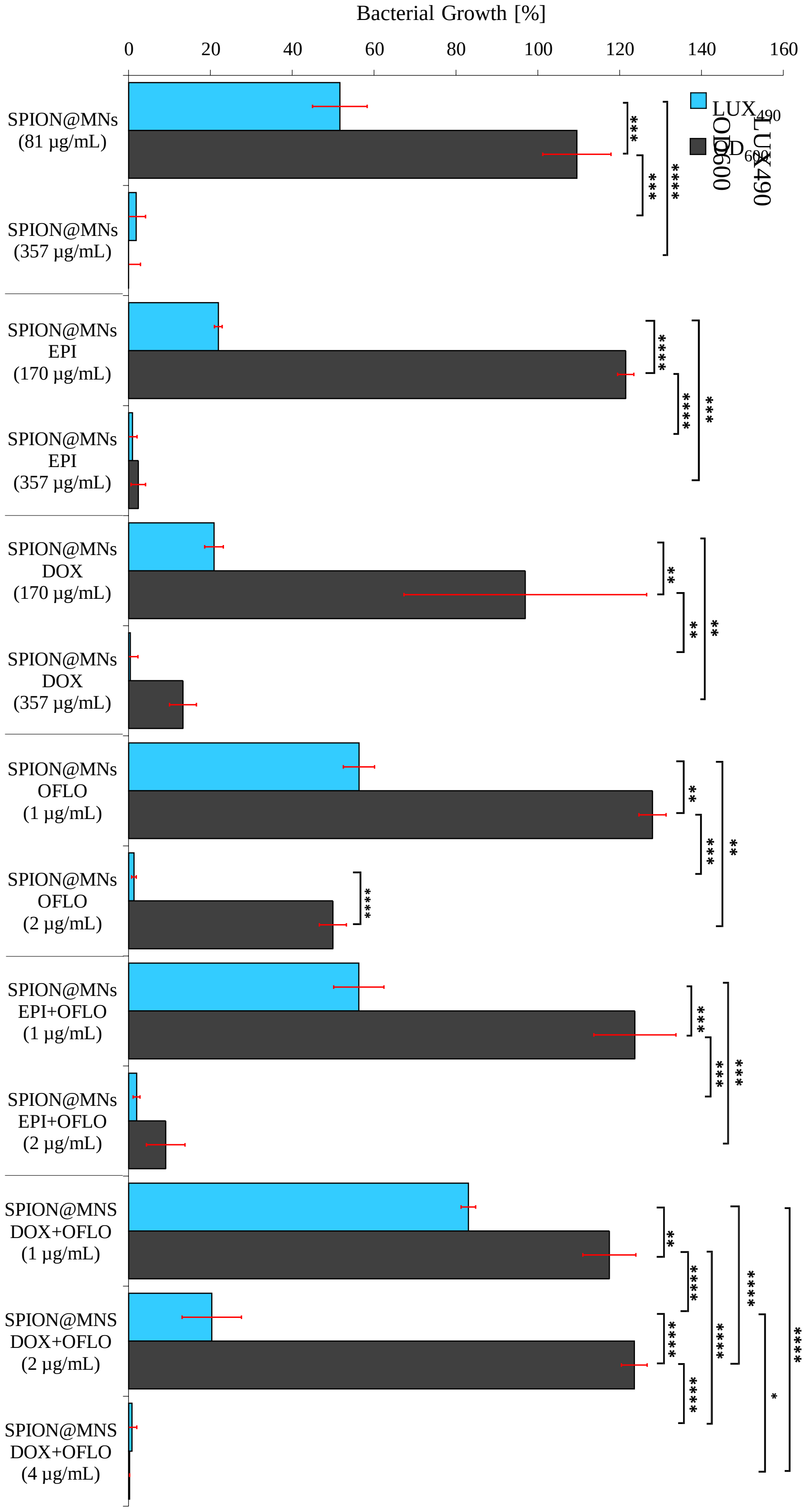

Figure S8. Statistical comparison of the obtained relative growth reduction, via OD_{600} 24 h-assaying the parental *E. coli* and LUX_{490} 8 h-assaying *E. coli* Lux, for all SPION@MNs systems (concentrations related to nanoparticle); statistically significant levels represented as * ≡ $p \leq 0.05$, ** ≡ $p \leq 0.01$, *** ≡ $p \leq 0.001$, **** ≡ $p \leq 0.0001$.

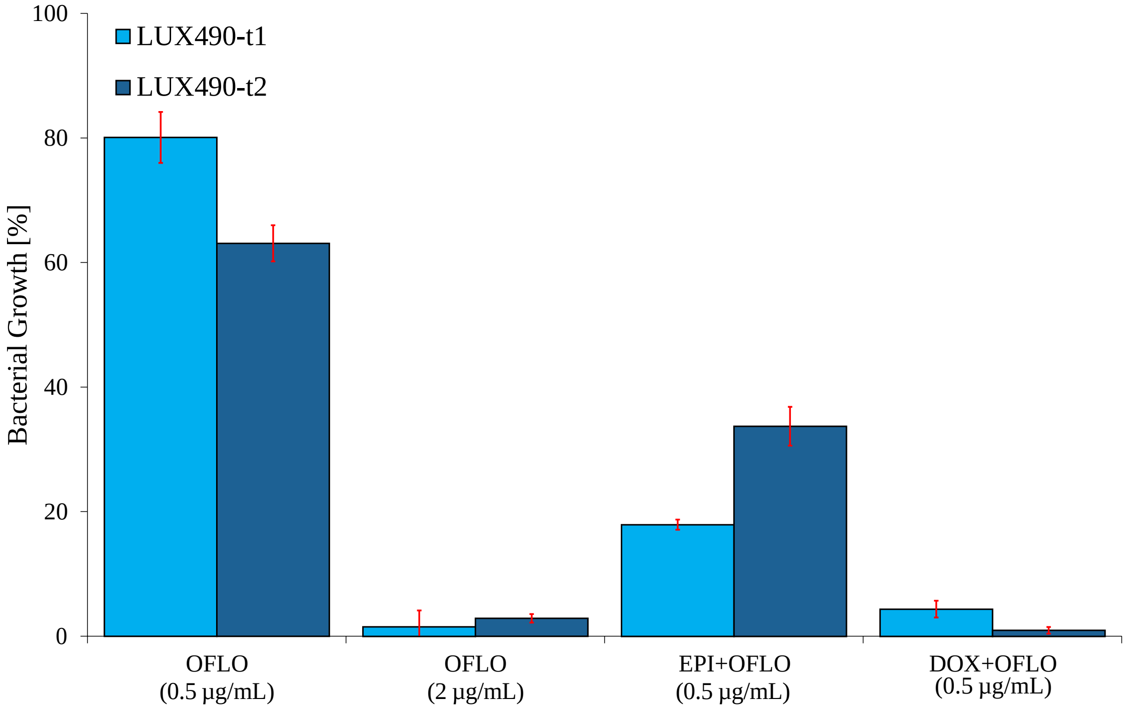

Figure S9. Statistical comparison of the MIC_{50}/MIC_{90} obtained, via LUX_{490} 8 h-assaying of *E. coli* Lux, for trial 1 (t1) and trial 2 (t2), of all OFLO-containing drug combinations; statistically significant levels represented as * ≡ $p \leq 0.05$, ** ≡ $p \leq 0.01$, *** ≡ $p \leq 0.001$, **** ≡ $p \leq 0.0001$.

References

1. Andreu, N.; Zelmer, A.; Sampson, S.L.; Ikeh, M.; Bancroft, G.J.; Schaible, U.E.; Wiles, S.; Robertson, B.D. Rapid in vivo assessment of drug efficacy against Mycobacterium tuberculosis using an improved firefly luciferase. *J. Antimicrob. Chemother.* **2013**, *68*, 2118–2127. https://doi.org/10.1093/jac/dkt155.
2. Bio-Rad Laboratories. MicroPulser™ Electroporation Apparatus Operating Instructions and Applications Guide. Bio-Rad: Hercules, CA, USA, 2016.
3. Addgene. Sequence Analyzer: pMV306G13+Lux. Available online: https://www.addgene.org/browse/sequence/12288/(accessed on 21 December 2021).
